# A specific inhibitor of ALDH1A3 regulates retinoic acid biosynthesis in glioma stem cells

**DOI:** 10.1038/s42003-021-02949-7

**Published:** 2021-12-21

**Authors:** Jianfeng Li, Silvia Garavaglia, Zhaofeng Ye, Andrea Moretti, Olga V. Belyaeva, Alison Beiser, Md Ibrahim, Anna Wilk, Steve McClellan, Alla V. Klyuyeva, Kelli R. Goggans, Natalia Y. Kedishvili, E. Alan Salter, Andrzej Wierzbicki, Marie E. Migaud, Steven J. Mullett, Nathan A. Yates, Carlos J. Camacho, Menico Rizzi, Robert W. Sobol

**Affiliations:** 1 Mitchell Cancer Institute, University of South Alabama, Mobile, AL 36604 USA; 2 Department of Pharmacology, College of Medicine, University of South Alabama, Mobile, AL 36604 USA; 3 Department of Pharmaceutical Sciences, University of Piemonte Orientale, Largo Donegani 2, 28100 Novara, Italy; 4 Department of Computational and Systems Biology, University of Pittsburgh, Pittsburgh, PA 15261 USA; 5 School of Medicine, Tsinghua University, Beijing, China; 6 Department of Biochemistry and Molecular Genetics, University of Alabama at Birmingham, Schools of Medicine and Dentistry, 720 20th Street South, Kaul 440B, Birmingham, AL 35294 USA; 7 Department of Chemistry, University of South Alabama, 6040 USA South Drive, Mobile, AL 36688 USA; 8 Department of Cell Biology, University of Pittsburgh, Pittsburgh, PA 15261 USA; 9 Present Address: Structural Plant Biology Laboratory, Department of Botany and Plant Biology, University of Geneva, 1211 Geneva, Switzerland

**Keywords:** Drug discovery, Structural biology, Cancer

## Abstract

Elevated aldehyde dehydrogenase (ALDH) activity correlates with poor outcome for many solid tumors as ALDHs may regulate cell proliferation and chemoresistance of cancer stem cells (CSCs). Accordingly, potent, and selective inhibitors of key ALDH enzymes may represent a novel CSC-directed treatment paradigm for ALDH^+^ cancer types. Of the many ALDH isoforms, we and others have implicated the elevated expression of ALDH1A3 in mesenchymal glioma stem cells (MES GSCs) as a target for the development of novel therapeutics. To this end, our structure of human ALDH1A3 combined with in silico modeling identifies a selective, active-site inhibitor of ALDH1A3. The lead compound, MCI-INI-3, is a selective competitive inhibitor of human ALDH1A3 and shows poor inhibitory effect on the structurally related isoform ALDH1A1. Mass spectrometry-based cellular thermal shift analysis reveals that ALDH1A3 is the primary binding protein for MCI-INI-3 in MES GSC lysates. The inhibitory effect of MCI-INI-3 on retinoic acid biosynthesis is comparable with that of ALDH1A3 knockout, suggesting that effective inhibition of ALDH1A3 is achieved with MCI-INI-3. Further development is warranted to characterize the role of ALDH1A3 and retinoic acid biosynthesis in glioma stem cell growth and differentiation.

## Abstract (web summary)

Using in silico modeling, Li et al. identify a selective inhibitor for aldehyde dehydrogenase isoform ALDH1A3, which is highly expressed in mesenchymal glioma stem cells and therefore a potential drug target. They solve the structure of the ALDH1A3/MCI-INI-3 inhibitor complex and find that the inhibitor is as efficient as ALDH1A3 depletion in preventing retinoic acid biosynthesis.

## Introduction

Cancer stem cells (CSCs) initiate and promote cancer development as well as promote therapeutic resistance and cancer recurrence via self-renewal, multi-lineage differentiation and tumorigenicity^1,2^. Aldehyde dehydrogenase (ALDH) activity is being considered as a potential prognostic marker for cancer since its increase in CSCs correlates with poor outcome for many solid tumors^3^. Further, it has been suggested that ALDHs regulate cell proliferation, cell survival and chemoresistance of CSCs^4–9^. Nineteen human genes in the ALDH superfamily have been identified since the first mammalian ALDH protein was purified^10^. These ALDH isozymes have diverse, and in some cases, overlapping functions that include preventing the accumulation of toxic aldehydes as well as the synthesis of vital biomolecules such as retinoic acid (RA), folate, and betaine^11–17^. The ALDH1A subfamily, comprised of isoforms ALDH1A1, ALDH1A2, and ALDH1A3, regulate RA signaling important for embryogenesis and development^18^. Following the first step of oxidation of Vitamin A (retinol) to retinaldehyde by members of the short-chain dehydrogenase/reductase superfamily of proteins, ALDH1A enzymes irreversibly convert retinaldehyde to RA—reviewed in^19^. RA can bind to retinoid receptors, regulating the transcription of more than 500 genes^20^. Although the ALDH isoforms 1A1, 1A2, and 1A3 recognize a common substrate, their expression pattern does not overlap entirely and thus may reflect a preference for specific substrates or biological endpoints.

The Aldefluor assay, developed for rapid analysis of the enzymatic activity of ALDHs in cells, is a well-established method to identify CSCs within a mixed population of heterogeneous tumor cells^21,22^. This is of value since it has been suggested that subpopulations of ALDH^High^ cancer cells show increased clonogenic potential, migration capacity, and tumor initiation as compared to ALDH^Low^ cancer cells^23^. However, it is not yet clarified if the high-ALDH activity observed in CSCs is the result of distinct or multiple ALDH isozymes^24^. Recently, it was found that of the 19 ALDH isoforms expressed in three different human cell lines (HEK293T, SUM159, and MDA-MB-231), nine isoforms are active in the Aldefluor assay, including ALDH1A1, ALDH1A2, ALDH1A3, ALDH1B1, ALDH2, ALDH3A1, ALDH3A2, ALDH3B1, and ALDH5A1^5^. Thus, it is critical to determine the dominant ALDH isoform in different cancer and tumor types.

High-ALDH activity in CSCs strongly suggests that the development of potent and selective inhibitors may represent a novel CSC-directed therapeutic potential in human cancers^18^. However, the development of selective inhibitors for each ALDH isoform is hindered by high sequence and structural homology. To-date, selective inhibitors have been developed for a few ALDH isoforms, including those of the ALDH1A family^25–30^. The isoform ALDH3A1 is selectively inhibited by the compound CB29 that binds in the aldehyde binding site with no measurable inhibition of the ALDH1A1, ALDH1A2, ALDH1A3, ALDH1B1 or ALDH2 isoforms at concentrations up to 250μM^31^; moreover, very recent studies also reported novel and potent inhibitors targeting ALDH1A3^25,32,33^. Based on the crystal structure of ALDH1A1 in a complex with NADH, selective inhibitors of human ALDH1A1 have also been identified^34^. Recently, we demonstrated that the ALDH1A3 isoform was highly and uniquely expressed in mesenchymal glioma stem cells (MES GSCs) and that RNA interference-mediated suppression of ALDH1A3 expression affected the growth of MES GSCs, suggesting ALDH1A3 as a potential target for glioblastoma treatment^3^. As a first step in the development of a small molecule inhibitor of ALDH1A3, we next reported the first crystal structure of human ALDH1A3 as a tetramer complexed with the cofactor NAD^+^ and the reaction product RA^35^.

To overcome the difficulty of the high sequence identity within the ALDH1A subfamily, we explored the structural differences among the isoforms to develop selective compounds targeting ALDH1A3. We note that such differences in the substrate access tunnel and/or in the catalytic pocket have been suggested to be responsible for the unique specificity that members of the ALDH family display for the different substrates^36^. This observation suggested that a high throughput in silico screening approach, targeting the substrate access tunnel and/or the catalytic pocket, may help to identify selective inhibitor candidates for ALDH1A3. Further validation of the selectivity of an ALDH inhibitor in cell lysates can also be achieved by thermal profiling of cellular proteomes^37,38^ followed by biochemical and cell-based analyses.

Here, we report on a selective inhibitor for ALDH1A3, identified by in silico modeling of the RA binding pocket based on our structure of human ALDH1A3 complexed with RA and NAD^+^^35^. In silico-derived lead compounds were biochemically validated as ALDH1A3-selective inhibitors and shown to block Aldefluor activity. The compound, MCI-INI-3, is a potent and selective inhibitor of recombinant human ALDH1A3, with greater than 140-fold selectivity for ALDH1A3 as compared to the closely related isoform ALDH1A1. Mass spectrometry-based cellular thermal shift analysis revealed that MCI-INI-3 binds selectively to the 1A3 isoform of ALDHs in cell lysates, and we show by analysis of a co-crystal of MCI-INI-3 and ALDH1A3 that the inhibitor binds in the active site. Further, the inhibitory effect of MCI-INI-3 treatment on RA biosynthesis is comparable to ALDH1A3 knockout cells, suggesting that the effective and selective inhibition of ALDH1A3 is achieved with MCI-INI-3.

## Results

### ALDH1A3 regulates proliferation, Aldefluor activity, and RA synthesis in mesenchymal glioma stem cells

**Fig. 1:**
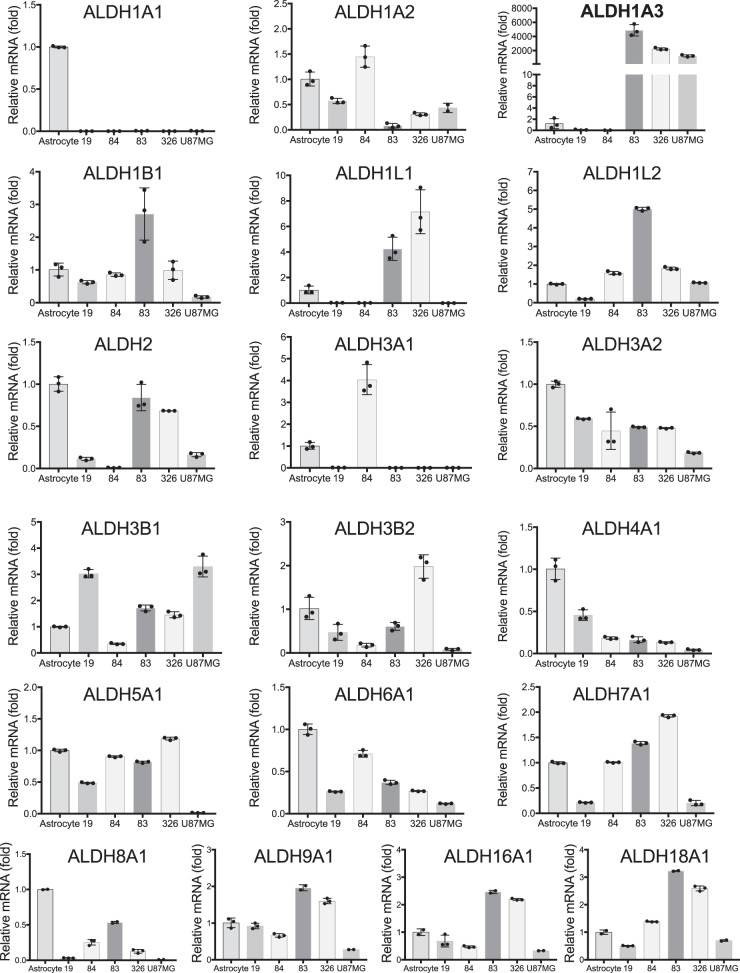
ALDH1A3 is the dominant ALDH isoform expressed in MES glioma stem cells. Quantitative RT-PCR analysis to measure the mRNA expression level of 19 ALDH isoforms in human astrocytes and GSC-19, GSC-84, GSC-83, GSC-326, and U87MG cells (*n* = 3 technical replicates).

In a previous study, we reported that there are two major subtypes of glioma stem cells (GSCs), proneural (PN) and mesenchymal (MES), and each present with distinct phenotypes and molecular signatures, in which ALDH1A3 mRNA expression is elevated in the MES subtype^3^. Here, we analyzed, via quantitative RT-PCR, the mRNA expression of all nineteen ALDH isoforms among the PN GSCs (GSC-19 and GSC-84), the MES GSCs (GSC-83 and GSC-326), the glioblastoma cell line U87MG and human astrocytes. ALDH1A3 is the predominant isoform, with expression in MES GSCs (GSC-83 and GSC-326) and U87MG cells greater than 1000-fold elevated, as compared to astrocytes. Conversely, we found only small variations in mRNA expression among the other ALDH isoforms across the cell lines (Fig. 1). Regarding the two other members of the ALDH1 subfamily, ALDH1A1 mRNA expression was not detected in either GSC-83 or GSC-326 cells, and ALDH1A2 showed minimal mRNA levels among all the cells evaluated (Fig. 1). We next expressed GFP tagged transgenes of ALDH1A1, ALDH1A2, and ALDH1A3 in GSC-83 cells to study the subcellular location of each of these ALDH1 family member proteins. As expected, the cellular localization of ALDH1A3, as well as the 1A1 and 1A2 isoforms, are uniquely cytosolic (Supplementary Fig. 1a).

**Fig. 2:**
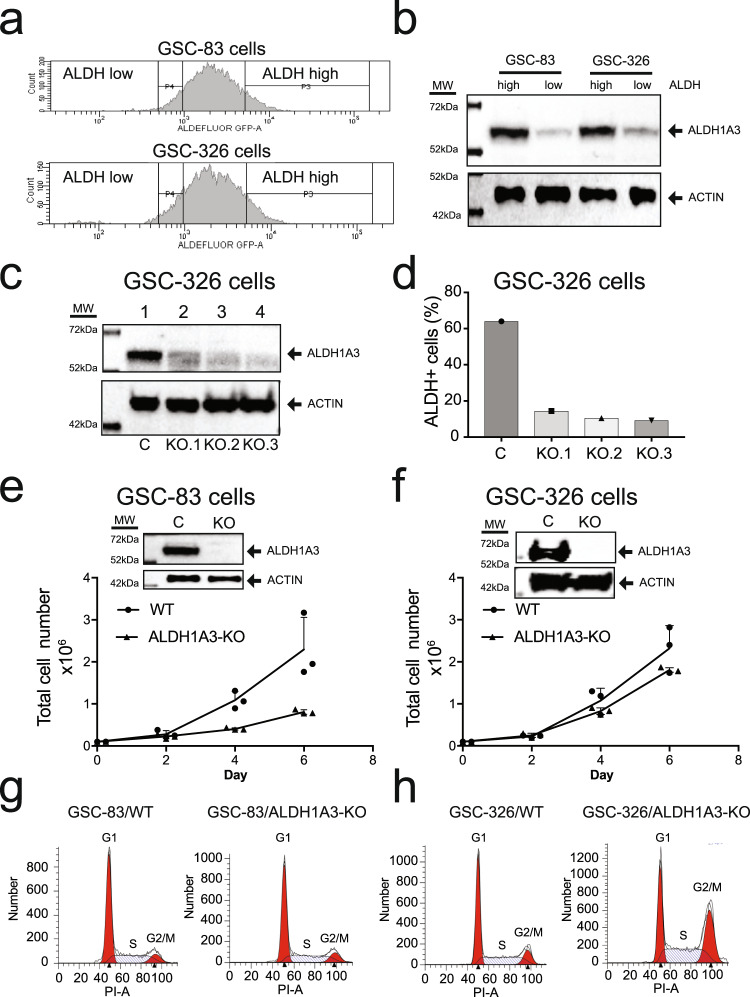
ALDH1A3 and proliferation of MES glioma stem cells. **a** The GSC-83 and GSC-326 MES cells with the top 10% ALDH activity and bottom 10% ALDH activity were sorted via Aldefluor FACS. **b** Immunoblotting analysis of ALDH1A3 protein in sorted cells with the top 10% ALDH activity and bottom 10% ALDH activity. **c** Immunoblotting analysis following acute depletion of ALDH1A3 (3 days after lentiviral transduction for expression of Cas9 and gRNA) in GSC-326 cells with three different guide RNAs (actin was used as the loading control). **d** After acute ALDH1A3 depletion, the ALDH activity of the GSC-326 cells were analyzed using the Aldefluor assay and plotted as the percentage of ALDH1^+^ cells for 3 individual ALDH1A3-KO pools. **e** The proliferation of GSC-83/ALDH1A3-KO cells normalized to the control cells (GSC-83, *n* = 3 technical replicates). An immunoblot confirming the loss of ALDH1A3 protein expression in the GSC-83/ALDH1A3-KO cells is shown in the inset. **f** The proliferation of GSC-326/ALDH1A3-KO cells normalized to the control cells (GSC-326, *n* = 3 technical replicates). An immunoblot confirming the loss of ALDH1A3 protein expression in the GSC-326/ALDH1A3-KO cells is shown in the inset. **g** Cell cycle analysis of GSC-83 (WT) and GSC-83/ALDH1A3-KO cells. **h** Cell cycle analysis of GSC-326 (WT) and GSC-326/ALDH1A3-KO cells.

As tumor stem cells, MES GSCs have a strong Aldefluor-mediated fluorescence signal, as we have shown in our previous report^3^. At least 9 of the 19 ALDH isoforms may be responsible for the positive Aldefluor signal^5^. However, given that ALDH1A3 is the highest expressed ALDH isoform in MES GSCs, we examined the contribution of ALDH1A3 to the overall enzyme activity of ALDH in two separate MES GSCs. The cells with the highest ALDH enzyme activity (top 10%, ALDH^High^) and those with the lowest ALDH enzyme activity (bottom 10%, ALDH^Low^) were isolated and analyzed for ALDH1A3 protein levels. In both MES GSC cell lines (GSC-83, GSC-326), ALDH activity (Fig. 2a) positively correlated to the ALDH1A3 protein level (Fig. 2b). We next established CRISPR/Cas9-mediated ALDH1A3 knockout (KO) MES GSC cell lines (GSC-83/ALDH1A3-KO and GSC-326/ALDH1A3-KO) to evaluate the impact of ALDH1A3 on the total ALDH enzyme activity of MES GSCs and to determine if the loss of ALDH1A3 affects MES GSC proliferation. To avoid potential compensation mechanisms from gene loss^39^, we first acutely depleted ALDH1A3 by lentiviral transduction, expressing Cas9 and either a control (non-targeting) gRNA or one of three ALDH1A3-specific gRNAs (see Supplementary Tables 1–3). We then evaluated the impact on ALDH1A3 protein expression and ALDH activity (Aldefluor) three (3) days after transduction. As shown, acute depletion of ALDH1A3 protein upon KO (Fig. 2c, lanes 2–4) reduced the ALDH activity (Fig. 2d) close to the levels seen in GSC-326 cells when they are treated with the pan-ALDH inhibitor DEAB (4.4%) (Supplementary Fig. 1b).

Because there was a small fraction of ALDH1A3 protein remaining in the acutely depleted ALDH1A3 cell pools (Fig. 2c), the addition of DEAB further reduced the Aldefluor fluorescence signal of ALDH1A3-KO cells to ~1%. Consistent with this result, a panel of GSC-326/ALDH1A3-KO stable single-cell clones, each harboring total loss of ALDH1A3 protein expression, showed strongly reduced Aldefluor fluorescence (1.6%; mean of 6 clones) (Supplementary Fig. 1c). To check if there is compensatory expression of ALDH1A1 or ALDH1A2 upon the loss of ALDH1A3, we also analyzed the expression of ALDH1A1 and ALDH1A2 in the six GSC-326/ALDH1A3-KO single-cell clones, as compared to the three GSC-326 (ALDH1A3 WT) control cells by microarray analysis. We did not detect any change in the expression of ALDH1A1 or ALDH1A2 in all GSC-326/ALDH1A3-KO clones (Supplementary Fig. 1d). We found that KO of ALDH1A3 only slightly impaired the proliferation of MES GSC-83 cells and GSC-326 cells, but each KO was viable with minimal difference over several weeks of culture (Fig. 2e, f). Cell cycle analysis revealed that though there was no observable cell cycle difference between GSC-83 and GSC-83/ALDH1A3-KO cells, there was a mild G2/M arrest in the GSC-326/ALDH1A3-KO cells (Fig. 2g). There were no observable differences in cell viability between the control and ALDH1A3-KO cells in either GSC cell lines (Supplementary Fig. 1e, f) so that the reduced proliferation by ALDH1A3 depletion may result from cell cycle arrest. Together, we validate ALDH1A3 as the dominant ALDH enzyme responsible for Aldefluor activity in MES GSC cells and that ALDH1A3 only minimally contributes to MES GSC proliferation.

**Fig. 3:**
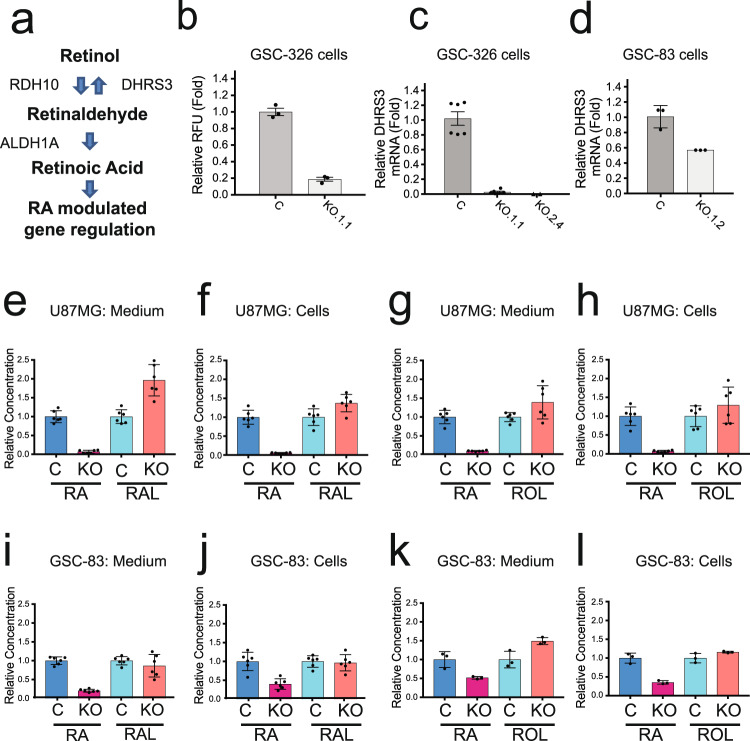
ALDH1A3 regulates RA synthesis in MES glioma stem cells. **a** Enzymes involved in the retinoic acid (RA) biosynthesis pathway. **b** GSC-326 control and GSC-326/ALDH1A3-KO cells were co-transfected with the reporter vector of RARE-Luciferase and Renila-Luciferase (the control vector). Forty-eight (48) hours after transfection, the luminescence was measured and normalized to the GSC-326 control cells as 1 (*n* = 3 technical replicates). **c** Quantitative RT-PCR analysis of the DHRS3 mRNA expression in GSC-326/ALDH1A3-KO single-cell clones. (Control: *n* = 6; KO.1.1, *n* = 6; KO.2.4, *n* = 2; technical replicates). **d** Quantitative RT-PCR analysis to evaluate DHRS3 mRNA expression in GSC-83/ALDH1A3-KO single-cell clones (*n* = 3, technical replicates). **e**–**h** Changes in metabolism of exogenously supplied retinoids in U87MG/ALDH1A3-KO cells. Retinol (ROL, 10 µM) (**e**, **f**) or retinoic acid (RA, 5 µM) (**g**, **h**) was added to U87MG/ALDH1A3-KO and control cells. After culturing, cells and culture medium were harvested separately, and retinoids were extracted and analysed by normal phase HPLC. The output of metabolites was compared with the control U87MG cell line normalized as 1 (*n* = 6, technical replicates). **i**–**l** Changes in metabolism of exogenously supplied retinoids in GSC-83/ALDH1A3-KO cells. The GSC-83/ALDH1A3-KO and control cells were treated with ROL (10 µM) (**i**, **j**; *n* = 6, technical replicates) or retinaldehyde (RAL, 5 µM) (**k**, **l**; *n* = 3, technical replicates) and the output of metabolites was compared with the control 83 MES GSC cell line normalized as 1.

Our previous structural analysis of human ALDH1A3 complexed with NAD^+^ and the reaction product RA^35^, was consistent with earlier work that had suggested retinaldehyde as the preferred substrate for both ALDH1A2 and ALDH1A3^40,41^. We therefore evaluated if ALDH1A3 was the dominant isozyme of the ALDH1A family involved in the RA biosynthesis pathway in MES GSCs (Fig. 3a). Using RARE-Firefly-Luciferase (RARE-LUC) as the RA reporter and Renila-Luciferase as the control, these two plasmids were co-transfected into MES GSCs (GSC-326 and GSC-326/ALDH1A3-KO cells). After co-transfection, the reporter system was validated in both cell lines as shown by an increase in luminescence from the RARE-LUC reporter following addition of exogenous RA as compared to the DMSO control (Supplementary Fig. 2a). The luminescence of the RARE-LUC reporter was strongly reduced in the GSC-326/ALDH1A3-KO cells, suggesting an overall decrease in the endogenous level of RA in the absence of ALDH1A3 (Fig. 3b).

The tightly regulated RA biosynthesis pathway includes the RA-inducible dehydrogenase reductase 3 (DHRS3) (Fig. 3a). This gene is induced by RA and provides regulatory control to the total level of RA by converting retinaldehyde (RAL) to retinol (ROL), thereby reducing the substrate for ALDH1 enzymes^42^. Consistent with this mechanism and in support of our proposal that ALDH1A3 is the predominant RA synthesizing enzyme in MES GSCs, the KO of ALDH1A3 also reduced the mRNA expression of DHRS3 in both MES GSC-326 and GSC-83 cell lines (Fig. 3c, d). When exogenous RA was added to the cells, mRNA expression of DHRS3 was upregulated in both control and ALDH1A3-KO cells, as measured by qRT-PCR, indicating RA-mediated modulation of DHRS3 mRNA expression (Supplementary Fig. 2b). We also checked the mRNA expression of DHRS3 in all 6 GSC-326/ALDH1A3-KO single cloned cells by microarray analysis. The mRNA expression of DHRS3 was reduced in each of the ALDH1A3-KO clones (Supplementary Fig. 2c), indicating specificity related to loss of ALDH1A3 expression.

To further evaluate the contribution of ALDH1A3 to the metabolism of retinoids, we modified the human glioblastoma cell line U87MG^43^ using the CRISPR/cas9 system to create U87MG/ALDH1A3-KO cells (Supplementary Fig. 2d). The cells lacking ALDH1A3 were treated with all-*trans*-ROL or all-*trans*-RAL, and the output of metabolites was compared with the control U87MG cell line. Upon treatment with ROL, U87MG cells readily produced RAL and RA. However, in the ALDH1A3 knockout cell line, the synthesis of RA was greatly reduced (10- to 30-fold in independent experiments) and was accompanied by an increase in RAL (Fig. 3e). A similar decrease in RA production by the U87MG/ALDH1A3-KO cells relative to control cells (U87MG) was observed when the cells were incubated with RAL, the immediate precursor of RA (Fig. 3e–h). These results are consistent with the disruption of the oxidation of RAL in ALDH1A3 knockout cells, highlighting the role of ALDH1A3 as the major retinaldehyde dehydrogenase in U87MG cells. These experiments were also repeated in the MES GSCs. Similarly, the biosynthesis of RA from ROL was strongly reduced in the ALDH1A3-KO GSC line (GSC-83/ALDH1A3-KO.1.2) in comparison to the control cells, GSC-83/CgRNA (~4- to 5-fold in independent experiments) (Fig. 3i–l). Incubation of the cells with RAL also revealed an approximately 2-fold decrease in RA output. This indicates that while ALDH1A3 is responsible for a greater proportion of retinaldehyde oxidation in GSCs, there appears to be a significant contribution to this step by other retinal dehydrogenases in MES GSCs.

### Structural comparison among different human ALDHs

**Fig. 4:**
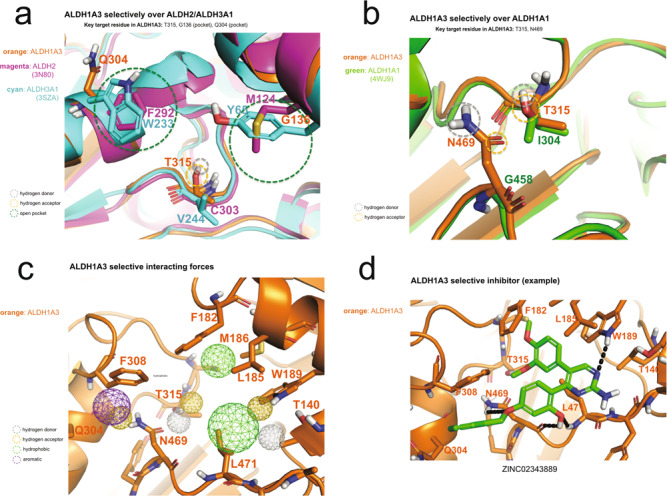
Structural comparison among different human ALDHs. **a** Relevant structural differences in the substrate access tunnel between ALDH1A3 and the isoforms ALDH2 and ALDH3A1. In the zoomed-in region, the green dotted line circles highlight the bulky residues that, in ALDH2 and ALDH3A1, decorate the substrate access tunnel and are painted in magenta and cyan, respectively. The structurally equivalent residues in ALDH1A3, T315 and G136, are in orange. Yellow and gray dotted line circles highlight the site of potential hydrogen bond donors and acceptors, respectively, and include T315, one of the two unique ALDH1A3 residues in the enzyme active site. **b** Structural comparison between ALDH1A1 and ALDH1A3 in the catalytic pocket revealed two major amino acid substitutions. Amino acid residues T315 and N469 in ALDH1A3 are replaced by I304 and G458, respectively, in ALDH1A1. Optimal structural superposition of ALDH1A3 (PDB code: 5FHZ) in orange and the two amino acids identified as contributors to selectivity are indicated: N469 and T315 in ALDH1A3 are drawn in orange and the structurally equivalent G458 and I304 in ALDH1A1 in green. Yellow and gray dotted line circles highlight the site of potential hydrogen bond donors and hydrogen bond acceptors, respectively. **c** The selective interacting force within the catalytic site of ALDH1A3. Yellow and gray dotted line circles highlight the site of potential hydrogen bond donors and acceptors, respectively. Green and violet dotted lines point up a zone with hydrophobic and aromatic possible interaction. **d** The example of the in silico design/screening of highly selective ALDH1A3 inhibitors.

The structural differences between the ALDH isozymes are critical for the design of a selective inhibitor due to the strong sequence similarities that exist among the ALDH isozymes. Although all ALDH1A isozymes can catalyze the conversion of RAL to RA, they show preferences for different retinaldehyde isomers, an observation that implies some diversity in the catalytic site and/or in the substrate access channel. ALDH1A3 shows a higher affinity for *all-trans* retinaldehyde^44^. The identification of residues critical for the reported specificity was carried out by superimposing the crystal structure of human ALDH1A3 with those of human ALDH1A1 (PDB code: 4WJ9), human ALDH2 (PDB code: 3N80) and human ALDH3A1 (PDB code: 3SZA) (Fig. 4). Relevant structural differences are indeed observed in the substrate access tunnel between ALDH1A3 and both ALDH2 and ALDH3A1. While ALDH2 and ALDH3A1 show two bulky amino acids located at the entrance and in the middle of the tunnel (M124 and F292 for ALDH2, and Y65 and W233 for ALDH3A1), the structurally equivalent positions are occupied by G136 and Q304 in ALDH1A3 (Fig. 4a) and G126 and H293 in ALDH1A1. Consequently, both ALDH2 and ALDH3A1 possess a narrow tunnel that is accessible only by small substrates such as acetaldehyde, while ALDH1A3 and ALDH1A1 exhibit a wider pocket that can admit a larger substrate such as retinaldehyde. On the other hand, when we focused our attention on the structural comparison between ALDH1A1 and ALDH1A3, we could not detect any obvious difference in the substrate access tunnel in the two isozymes. However, two major amino acid substitutions can be observed within the catalytic pocket, in the immediate vicinity surrounding the conserved catalytic cysteine. Amino acid residues T315 and N469 in ALDH1A3 are replaced by I304 and G458, respectively, in ALDH1A1 (Fig. 4b). Remarkably, ALDH1A3 appears to have two peculiar and specific amino acid substitutions in its catalytic site, with two hydrophilic residues that can establish hydrogen bonds with a ligand/inhibitor. Such a peculiar feature was indeed the key structural determinant for the in silico design/screening of highly selective ALDH1A3 inhibitors (Fig. 4c, d).

### Rational discovery of a selective ALDH1A3 inhibitor by in silico screening

As suggested in Fig. 4, we proceeded to design several chemotypes to search for selective inhibitors of ALDH1A3. Specifically, a hydrogen bond acceptor was designed to target T315; an aromatic chemotype was placed to form π-π stacking with F308; and a hydrophobic moiety was used to fill the cavity near G136 (Fig. 4c). We implemented this design in ZincPharmer^45,46^ (http://zincpharmer.csb.pitt.edu/), a pharmacophore-based drug discovery tool that screens more than 26 million commercially available compounds from the ZINC database^47^. Compounds that matched the stipulated design were further minimized with SMINA^48^. The filtered compounds were clustered, based on chemical scaffolds. The diversity of the scaffolds in return guided us to refine the designs. Several rounds of screening-minimizing-clustering were carried out to finally converge on twenty-seven (27) candidate compounds (Supplementary Table 4, Supplementary Fig. 3)^49^.

### Biochemical characterization of ALDH1A1 and ALDH1A3

**Fig. 5:**
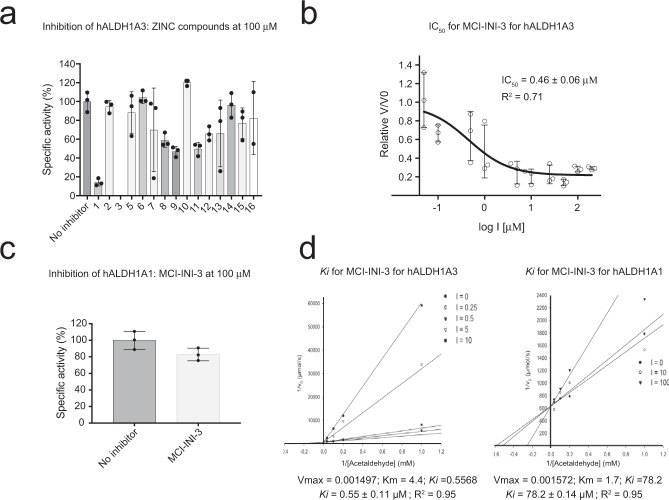
Biochemical characterization of MCI-INI-3 inhibition of ALDH1A1 and ALDH1A3. **a** Histogram representation of the specific residual enzymatic activity of ALDH1A3 in the presence of 100 µM inhibitor concentration of the selected inhibitor compound and in absence of any inhibitor (first column on the left; *n* = 3, technical replicates). **b** Graphical representation of MCI-INI-3 IC_50_ for ALDH1A3 (*n* = 3, technical replicates). **c** Histogram representation of the specific residual enzymatic activity of ALDH1A1 in the presence of 100 µM MCI-INI-3 and with no inhibitor. ALDH1A1 retains 92% of its specific activity with 100 µM MCI-INI-3 (*n* = 3, technical replicates). **d** Steady-state enzyme kinetics with MCI-INI-3 for ALDH1A3 and ALDH1A13. The experimental procedure is detailed in the Material and Methods section and the reported values represent the average of three independent experiments. Each line represents linear regression analysis of the reciprocal of average simulated rates of product formation for different substrate concentrations as a function of inhibitor concentration. IC_50_ and *K*_*i*_ values are reported.

Pure and active recombinant ALDH1A3 and ALDH1A1, produced with excellent yield as detailed in the Materials and Methods section, were used for enzymatic and structural investigations (Supplementary Fig. 4). Compounds identified as potential ALDH1A3 inhibitors by in silico screening were tested against the enzyme at a fixed concentration (100 µM). Out of the initial 16 selected small molecules (MCI-INI-1 through MCI-INI-16), only MCI-INI-4 could not be tested due to its poor solubility. All the remaining compounds (Supplementary Table 4, Supplementary Fig. 3) were initially scored according to the percentage of residual ALDH1A3 enzymatic activity, and MCI-INI-3 emerged as the most potent (Fig. 5a) for which an IC_50_ of 0.46 ± 0.06 μM was subsequently determined (Fig. 5b). As the major aim of our work was to develop a potent and selective ALDH1A3 inhibitor, MCI-INI-3 was then tested against ALDH1A1 (100 µM), showing only a marginal effect with the enzyme retaining 92% of its enzymatic activity (Fig. 5c). At this point, a complete determination of the Michaelis-Menten kinetics was performed that revealed MCI-INI-3 to be a competitive inhibitor for the aldehyde substrate, with *K*_*i*_ values of 0.55 ± 0.11 μM for ALDH1A3 and 78.2 μM ± 14.4 μM for ALDH1A1 (Fig. 5d). Therefore, we can conclude that MCI-INI-3 is a potent and selective ALDH1A3 inhibitor showing a selectivity index toward the ALDH1A1 isozyme of about 140.

### Structural analysis of the ALDH1A3/MCI-INI-3 complex

**Fig. 6:**
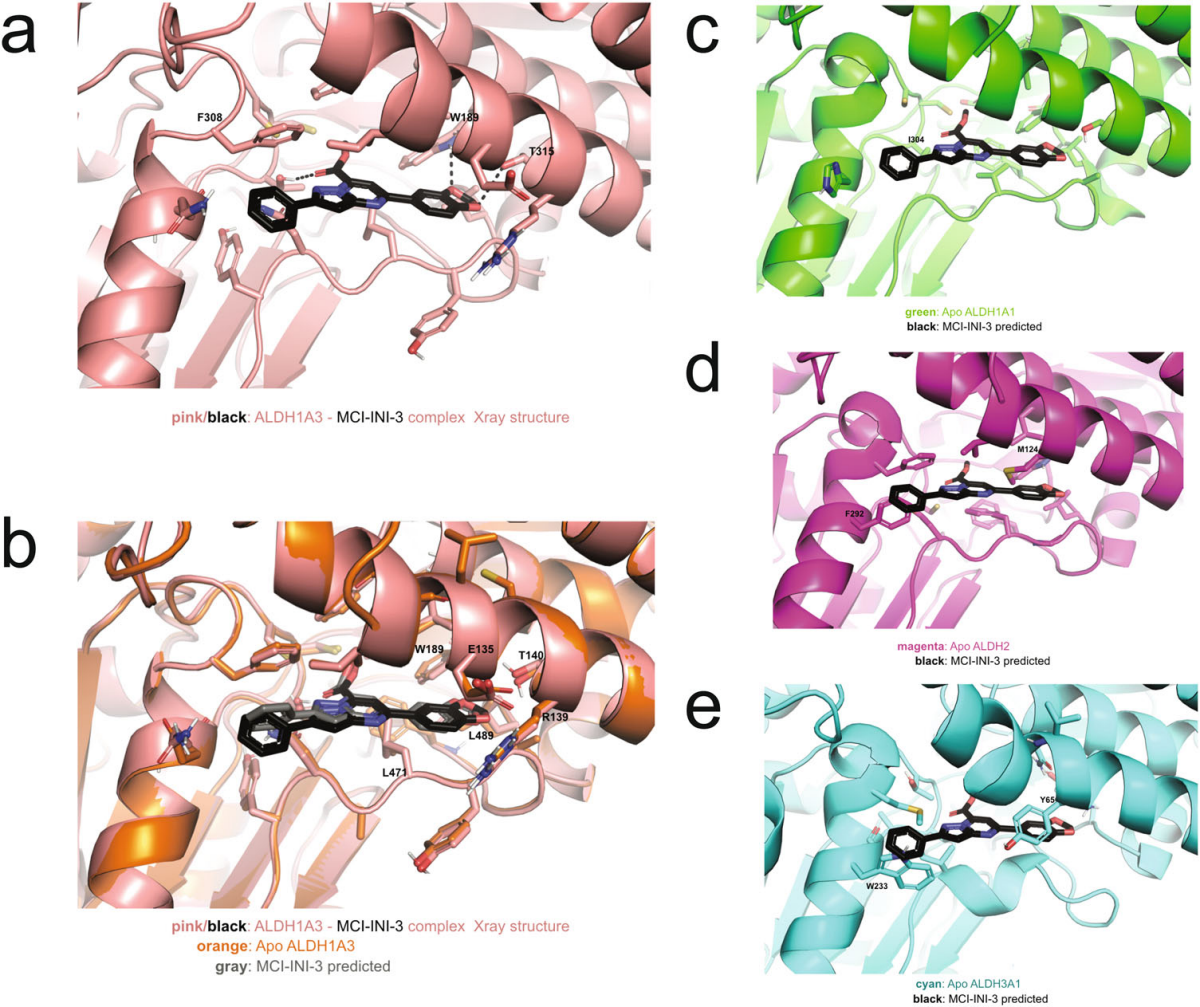
Discovery of a selective ALDH1A3 inhibitor by in silico screening. **a** X-ray crystal structure of human ALDH1A3 (in pink) in complex with MCI-INI-3 (in black) (PDB code 6TGW). Key interacting residues are shown in sticks. Dashed lines represent hydrogen bonds. **b** Superposition of the ALDH1A3 crystal structure (orange, PDB code: 5FHZ) with the predicted pose of MCI-INI-3 (in gray) and the co-crystal structure of ALDH1A3 with MCI-INI-3. **c**–**e** Superposition of the MCI-INI-3 inhibitor as observed in the ALDH1A3 co-crystal to the structure of ALDH1A1 (in green, PDB code: W4J9), ALDH2 (in magenta, PDB code: 3N80) and ALDH3A1 (in cyan, PDB code: 3SZA), respectively. The dashed circles highlight the conflicts of MCI-INI-3 with other ALDHs. Images were prepared using the program PyMol.

While most compounds showed some degree of inhibition, activity and selectivity experiments suggested that MCI-INI-3 had the best biochemical profile for further studies (Fig. 5). Hence, we obtained the co-crystal of MCI-INI-3 and ALDH1A3 (PDB code: 6TGW) (Fig. 6a). The three-dimensional structure of human ALDH1A3 in a complex with MCI-INI-3 was determined at a resolution of 2.8 Å. The final model contains four identical protein chains per asymmetric unit, arranged in a tetramer, a total of 257 solvent molecules and 4 inhibitor molecules (Table 1). The co-crystal validated our in silico approach by yielding a structure that is very similar to that predicted by molecular docking onto the ALDH1A3 crystal structure (PDB code: 5FHZ, complex with NAD^+^ and RA). As predicted, the phenyl group of MCI-INI-3 formed a π-π stacking interaction with F308, and the carboxyl group formed a hydrogen bond with T315. The 1,3-benzodioxol-5-yl group was deeply buried in the enzyme hydrophobic pocket formed with E135, R139, T140, W189, L471, and L489 (Fig. 6b). In addition, two weak hydrogen bonds between T140 and W189 and MCI-INI-3 further stabilized the complex (Fig. 6a,b). The superposition of the co-crystal to other ALDHs further rationalized the observed selectivity. Figure 6c shows no hydrogen donor residue in ALDH1A1 to match the carboxyl group of MCI-INI-3. T315 in ALDH1A3 is I304 in ALDH1A1 (Fig. 4b), which would severely penalize MCI-INI-3 binding to ALDH1A1. In Fig. 6d, the hydrophobic pockets that buried the carboxyl and 1,3-benzodioxol-5-yl groups of MCI-INI-3 in ALDH1A3 were blocked with F292 and M124 in ALDH2. The carboxyl group also had no match in the absence of hydrogen bond donors. Finally, as shown in Fig. 6e, the two hydrophobic pockets were buried by W233 and Y65, respectively, in ALDH3A1. In summary, the targeting of T315 and the two hydrophobic pockets near the substrate access tunnel is the structural basis of the compound’s selectivity for ALDH1A3 over other ALDHs.

**Table 1:** Data collection and refinement statistics.

	6TGW
Data collection	
Space group	P2_1_2_1_2_1_
Cell dimensions	
*a*, *b*, *c* (Å)	81.03, 158.48, 168.65
α, β, γ (°)	90.00, 90.00, 90.00
Resolution (Å)	47.51–2.8
*R*_merge_	0.094 (0.374)
*I* / σ*I*	7.0 (2.1)
Completeness (%)	97.2 (92.4)
Redundancy	2.0 (1.8)
Refinement	
Resolution (Å)	47.51–2.8
No. reflections	52,790 (4947)
*R*_work_/*R*_free_	0.1948 (0.2731)/0.2599 (0.3457)
No. atoms	
Protein	1913
Ligand/ion	257
Water	288
*B*-factors	
Protein (Å^2^)	25.49
Ligand/ion (Å^2^)	38.73
Water (Å^2^)	18.94
R.m.s. deviations	
Bond lengths (Å)	0.009
Bond angles (°)	1.13

**Fig. 7:**
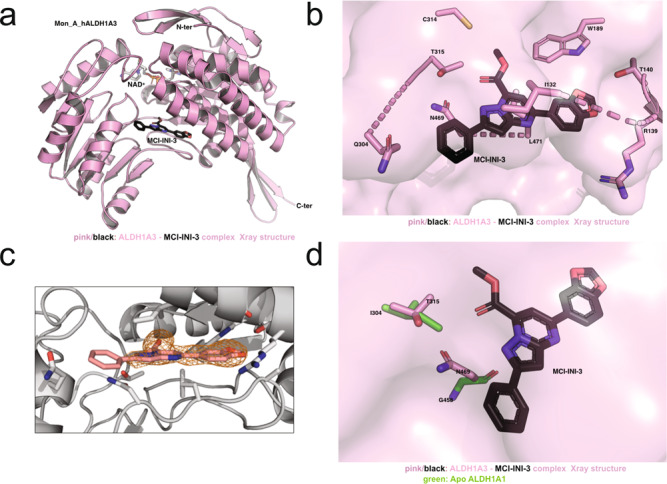
Crystal structure analysis of ALDH1A3 in complex with MCI-INI-3. **a** Ribbon representation of the ALDH1A3 monomer structure. The ligands NAD^+^ and MCI-INI-3 are shown as gray and black sticks. **b** Zoom-in of MCI-INI-3 binding site. The inhibitor is shown as black sticks and the residues defining the binding pockets as pink sticks. **c** Omit Fo–Fc electron density map covering MCI-INI-3. The omit electron density map is shown in orange contoured at 2.5 standard deviations. **d** Superposition of the ALDH1A3 and ALDH1A1 structural elements responsible for MCI-INI-3 selectivity for ALDH1A3: N469 and T315 in ALDH1A3 (pink sticks) superimposed to the structurally equivalent G458 and I304 in ALDH1A1. MCI-INI-3 is shown in black sticks. All the images were prepared using the program PyMol.

Inspection of the crystal structure also revealed that the inhibitor binds to the enzyme active site, overlapping the previously described retinaldehyde binding pocket of ALDH1A3^35^ that extends from the protein surface to the catalytic C314 (Fig. 7a). This structural observation therefore fully explains the observed competitive behavior of the inhibitor. The molecule (MCI-INI-3) establishes multiple interactions with protein residues (Fig. 7b). In particular, the ester group of MCI-INI-3 pointed towards the catalytic cysteine at a distance of about 5 Å and its benzodioxole moiety established several contacts with the protein environment, including an H-bond with W189 and Van der Waals contacts with T140 and R139. The phenyl group of MCI-INI-3, which makes a π-π stacking interaction of variable quality with F308, sits close to the protein surface and appears to be rather flexible, being poorly visible in the omit electron density map (Fig. 7c). With respect to the specific selectivity that MCI-INI-3 shows for ALDH1A3, our structural data revealed that its pyrazolopyrimidine ring contacts N469 (N12-NH_2_ distance of about 4 Å) and its ester group interacts with T315 (O4-OH distance of about 4 Å), the two amino acids that distinguish the 1A3 isozyme active site. Such stabilizing interactions would simply be impossible in ALDH1A1 where the two structurally equivalent positions are occupied by I304 and G458 (Fig. 7d), residues that cannot establish electrostatic interactions with the hydrophilic portions of the inhibitor.

### Differential mass spectrometry and cellular thermal shift analysis

**Fig. 8:**
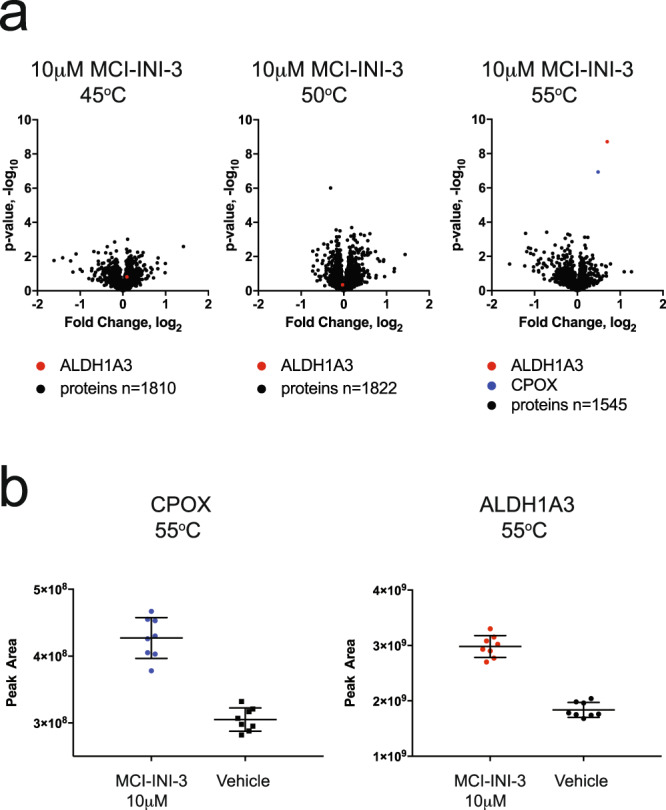
Selective binding of MCI-INI-3 to ALDH1A3 in glioma stem cell lysates. **a** Scatter plots of statistical significance versus the change in relative protein abundance for 1810, 1822, and 1545 protein groups were determined by label-free differential mass spectrometry for cellular thermal stability experiments conducted at 45, 50, and 55 °C, respectively. Quantification of unique protein groups was accomplished with the MaxQuant software program and outliers were removed with peptide occupancy filtering. **b** Box and whisker plots of *ALDH1A3* and *CPOX* protein abundance for vehicle and MCI-INI-3 treated (10 μM) lysates following heating at 55 °C and centrifugation at 25,000 × *g*. Each of the eight technical replicates (*n* = 8) was digested with trypsin and subjected to LC-MS/MS based proteomic analysis.

To determine if MCI-INI-3 binds to ALDH1A3 in cell lysates (GSC-326), we employed an approach combining label-free differential mass spectrometry and cellular thermal shift analysis, which allows for the identification of proteins that bind small molecules in a whole-cell lysate^37,38^. Cell lysates from GSC-326 cells were incubated with MCI-INI-3 at 18x the *K*_*i*_ value (10 μM; Ki = 0.55 μM) (Fig. 8a), and the thermal shift analysis and label-free differential mass spectrometry yielded volcano plots of statistical significance versus magnitude of change in relative protein abundance for vehicle and MCI-INI-3 treated lysates following heating and centrifugation. MCI-INI-3 was found to induce a selective shift in the thermal stability of ALDH1A3 at 55 °C, with a *p* value of 2.0 × 10^−9^. Mitochondrial oxygen-dependent coproporphyrinogen-III oxidase (CPOX) exhibited the next most significant thermal shift at 55 °C with a *p* value of 1.7 × 10^−7^. In total, more than 1500 proteins, including six isoforms of ALDH (ALDH16A1, ALDH7A1, ALDH9A1, ALDH2, ALDH1B1, ALDH18A1), were probed at three experimental temperatures (45, 50, and 55 °C) and only two proteins (ALDH1A3 and CPOX) were found to have a strong interaction with MCI-INI-3. Mass spectrometry-based measurement of the decrease in the relative abundance of ALDH1A3 and CPOX in technical replicates (*n* = 8) of vehicle and compound treated soluble GSC lysates following heat treatment at 55 °C and centrifugation at 25,000 × *g* is shown (Fig. 8b). To exclude the possibility that binding and inhibition of CPOX may be related to the cellular effects observed following MCI-INI-3 treatment, we developed U87MG/CPOX-KO cells (Supplementary Fig. 5a). It is noted however that cell proliferation was not affected by the loss of CPOX (Supplementary Fig. 5b).

### Small molecule inhibition of ALDH1A3 regulates Aldefluor activity and RA synthesis in MES glioma stem cells

**Fig. 9:**
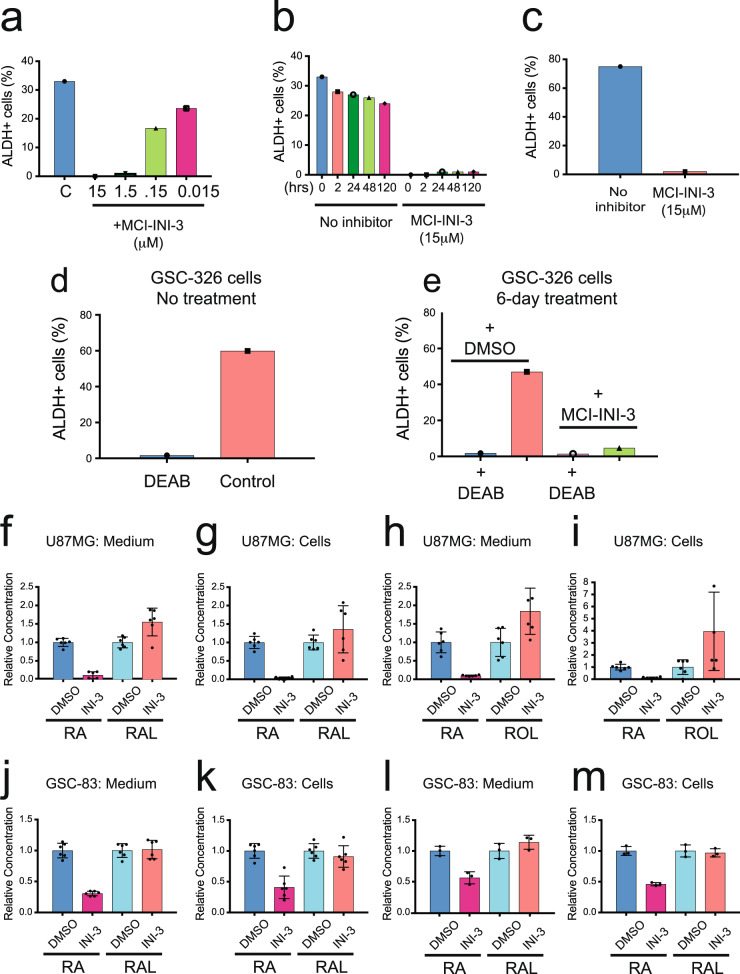
MCI-INI-3 inhibits ALDH activity and affects RA synthesis in cells. **a** Inhibition of ALDH activity in U87MG cells by MCI-INI-3 with different concentrations measured with the Aldefluor assay and plotted as the percentage of ALDH1^+^ cells. **b** Inhibition of ALDH activity in U87MG cells by MCI-INI-3 (15 µM) at different time points measured with the Aldefluor assay and plotted as the percentage of ALDH1^+^ cells. **c** U87MG cells were sorted using Aldefluor-FACS to enrich the ALDH high population to 75%. The enriched cells were then treated with MCI-INI-3 (10 μM) for 15 min. The ALDH activity was assayed by the Aldefluor assay and plotted as the percentage of ALDH1^+^ cells. **d** The ALDH activity of GSC-326 cells was analyzed using the Aldefluor assay without treatment and plotted as the percentage of ALDH1^+^ cells. DEAB was used as the negative control. **e** After a 6-day treatment with MCI-INI-3 (15 µM) or DMSO, the ALDH activity of GSC-326 cells were analyzed using the Aldefluor assay and plotted as the percentage of ALDH1^+^ cells. DEAB was used as the negative control. DMSO or MCI-INI-3 (15 µM) was added at the same time as ROL (10 µM) (**f**, **g**) or RAL (5 µM) (**h**, **i**) to U87MG cells. After culturing, cells and culture medium were harvested separately, and retinoids were extracted and analyzed by normal phase HPLC. The output of metabolites was compared with the control U87MG cell line, normalized as 1 (*n* = 6, technical replicates). DMSO or MCI-INI-3 (15 µM) was added at the same time as ROL (10 µM) (**j**, **k**; *n* = 6, technical replicates) or RAL (5 µM) (**l**, **m**; *n* = 3, technical replicates) to GSC-83/ALDH1A3-KO.1.2 and control cells (GSC-83). After culturing, cells and culture medium were harvested separately, and retinoids were extracted and analyzed by normal phase HPLC. The output of metabolites was compared with the control GSC-83 cell line, normalized as 1.

Given the strong and selective inhibition of ALDH1A3 by MCI-INI-3 using purified proteins, we next evaluated MCI-INI-3-mediated inhibition of ALDH1A3 in intact cells using the Aldefluor activity assay. For U87MG cells, non-treated cells showed 33% Aldefluor-positive cells, whereas MCI-INI-3 treatment reduced Aldefluor reactivity to 1% (1.5 µM) and was reduced completely at higher doses (15 µM) (Fig. 9a, Supplementary Fig. 6a), with loss of Aldefluor activity for as much as 120 h (Fig. 9b, Supplementary Fig. 6b). As confirmation, we FACS sorted U87MG cells and enriched the Aldefluor-positive cells to 75%. Within 15 min of MCI-INI-3 treatment (10 µM) at 37 °C, the level of Aldefluor-positive cells was reduced to 2% (Fig. 9c, Supplementary Fig. 6c). We then evaluated ALDH activity in the MES cell line GSC-326 before and after MCI-INI-3 treatment (6 days) to check the long-term inhibition of ALDH activity by MCI-INI-3 in cells. After 6 days, control cells treated with DMSO showed little, if any, change in the percentage of ALDH positive cells, as compared to the non-treated MES GSCs (44.7% vs 55.9%). However, MCI-INI-3 treatment (15 µM) strongly reduced the level of Aldefluor-positive cells, with a 10-fold reduction to 4.7% (Fig. 9d, e and Supplementary Fig. 6d–f). Overall, we show that the small molecule ALDH1A3 inhibitor MCI-INI-3 inhibits ALDH activity in cells, with strong inhibitory activity lasting at least 6 days.

Further, to test the effect of this ALDH1A3-specific inhibitor on RA production, control U87MG cells and GSCs were treated with retinoids in the presence of MCI-INI-3 (15 µM) or vehicle (DMSO). The level of RA produced by U87MG cells from either ROL or RAL in the presence of MCI-INI-3 was dramatically decreased in comparison to the cells treated with vehicle (~10 to 30-fold in independent experiments) (Fig. 9f–i). Like the ALDH1A3-KO cells, the effect of MCI-INI-3 on RA biosynthesis in the GSCs was less pronounced than in U87MG cells, leading to a ~3-fold decrease in RA production from ROL, and a 2-fold decrease in RA production from RAL (Fig. 9j–m). The effect of the inhibitor treatment on RA biosynthesis by these two cell lines is comparable to that of ALDH1A3 knockout in the same cells, suggesting that the effective inhibition of ALDH1A3 is achieved by treatment with MCI-INI-3.

### ALDH1A3 and glioma stem cell proliferation

The loss of expression of ALDH1A3, via CRISPR/cas9-mediated gene KO in both the GSC-83 and GSC-326 cells, had only a small impact on cell proliferation (Fig. 2e, f). However, all the GSC/ALDH1A3-KO cells developed to-date are viable, raising the question as to the connection between ALDH1A3 activity and cell viability. Given that MCI-INI-3 treatment of GSCs results in a strong block to ALDH activity (Supplementary Fig. 6) and a loss of RA biosynthesis (Fig. 3), we evaluated if MCI-INI-3 treatment impacts the growth of MES GSCs. As shown (Supplementary Fig. 7a), MCI-INI-3 treatment (15 μM) reduced cell proliferation of both the GSC-326 and GSC-83 MES cell lines. However, MCI-INI-3 also reduced cell proliferation of two PN-type GSCs, GSC-19 and GSC-84, neither of which express ALDH1A3 (Supplementary Fig. 7b). Further, we completed a more detailed cell viability analysis of both DEAB and MCI-INI-3 by treating GSC-326 and GSC-326/ALDH1A3-KO cells with a broad-range dose-response of each compound (Supplementary Fig. 7c). We determined the IC_50_ for both compounds for the WT cells (MCI-INI-3, 26.21 μM; DEAB, 23.78 μM) and for the ALDH1A3-KO cells (MCI-INI-3, 28.21 μM; DEAB, 23.47 μM). While both compounds lead to a loss of cell viability, neither DEAB nor MCI-INI-3 has an ALDH1A3-specific impact on the proliferation of MES GSCs.

## Discussion

In recent years, it has been widely accepted that CSCs are the major driving force responsible for tumor recurrence and therapy resistance. Therefore, targeting CSCs to remove the re-initiating capability of tumor cells may be promising therapeutically. High-ALDH activity, as measured by the Aldefluor assay, is one hallmark biomarker for CSCs. However, given that there are 19 ALDH isozymes in the human ALDH superfamily, the major ALDH isoform(s) responsible for Aldefluor activity may be different depending on cancer type and tissue/cell of origin. For example, upregulation of ALDH1A3 is associated with distant metastasis, poor disease-free survival and overall survival in breast cancer^50^. Elevated ALDH1A1 expression is associated with poor 5-year overall survival for ovarian cancer patients^51^ as well as poor response to platinum-based therapy in patients with high-grade ovarian serous carcinoma^52^. ALDH7A1 plays an important role in prostate cancer bone metastasis^53^ and ALDH1B1 may be involved in colon cancer tumorigenesis via the Wnt/β-catenin, Notch and PI3K/Akt signaling pathways^54^. Thus, identification of the dominant ALDH isozyme in specific tumor types is critical for precise CSC targeting. In this study, we investigated the contribution of ALDH1A3 regarding Aldefluor activity in MES GSCs. The top 10% ALDH^High^ MES GSCs showed much higher ALDH1A3 protein levels as compared to the bottom 10% (ALDH^Low^ MES GSCs), and knockout of ALDH1A3 abolished Aldefluor activity of MES GSCs. However, the depletion of ALDH1A3 only minimally slowed proliferation of MES GSCs. These findings point to ALDH1A3 as the major contributor of Aldefluor activity and may contribute only a minor role, if any, in MES GSC proliferation.

Because the ALDH1 gene family plays an important role in the conversion of retinaldehyde into RA to modulate the expression of target genes, we also investigated the importance of ALDH1A3 for RA synthesis in MES GSC cells. Here we show that depletion of ALDH1A3 resulted in a strong decrease in the production of RA in glioma-derived cells (U87MG) and MES GSCs. Further, when exogenous ROL or RAL was added as the upstream substrates to the ALDH1A3-KO or control cells (U87MG and GSC-83 cells), the RA concentration within the cells or in growth medium was strongly decreased when ALDH1A3 was absent. These results indicate that ALDH1A3 plays a major role in RA biosynthesis in MES GSCs. Taking the results of the Aldefluor assay and the RA synthesis experiment together, our data provide evidence that ALDH1A3 is the functionally dominant ALDH isozyme in MES GSCs. As such, the development of small molecular inhibitors targeting ALDH1A3 may provide a potential for targeted therapy for glioblastoma patients with tumors harboring a MES signature^55^.

The major challenge for the development of inhibitors for individual ALDH isozymes is the high sequence similarity among those isozymes. There is more than 40% sequence identity among the 19 isozymes of the ALDH superfamily and more than 60% sequence identity among the members of each subfamily^18^. This lack of selectivity of current inhibitors makes it hard to avoid inhibiting one ALDH isozyme without affecting the others. For example, although diethylaminobenzaldehyde (DEAB) was reported as a specific inhibitor for the ALDH1 isoform, it is an excellent substrate for ALDH3A1. DEAB is also a substrate for ALDH1A1, ALDH1A3, ALDH1B1, and ALDH5A1^56^. However, the turnover rates are so low that it may be a competent inhibitor for those isozymes. In addition, DEAB behaves as a covalent inhibitor for ALDH1A2 and ALDH2^56^. We hypothesized therefore that inhibitor selectivity may be improved with a focus on the differences between the critical residues for ALDH activity. We carefully compared the structure of ALDH1A3 we reported recently^35^ to that of ALDH1A1, the isozyme in the ALDH1 family with the greatest homology to ALDH1A3. Even with a high degree of structural similarity (r.m.s.d. of 0.96 Å and 71% amino acid identity), a few important residues for substrate selectivity in the catalytic pockets are unique. There is an asparagine at position 469 and a threonine at position 315 in ALDH1A3, but those respective residues are replaced with a glycine and an isoleucine in ALDH1A1. These critical structural differences between ALDH1A1 and ALDH1A3 made it possible to conduct a high-throughput in silico screening analysis, probing the catalytic pocket of ALDH1A3 against the ZINC database^47^. Twenty-seven compounds with the highest affinity scores were listed as possible selective inhibitors of ALDH1A3. In the ALDH1A3 in vitro enzymatic activity assay, 4 compounds showed some measure of ALDH1A3 inhibition, with MCI-INI-3 being the most potent. Importantly, as was predicted, even at 100 μM, MCI-INI-3 is a poor inhibitor of ALDH1A1. A Michaelis-Menten kinetics study further demonstrated that MCI-INI-3 is a competitive inhibitor for the aldehyde substrate, with *K*_*i*_ values of 556 nM for ALDH1A3 and 78 µM for ALDH1A1. We further demonstrated selectivity of MCI-INI-3 against ALDH1A3 ex vivo by measuring its thermostability with ALDH1A3 using differential mass spectrometry combined with cellular thermal shift analysis. The most significant protein bound by MCI-INI-3 is ALDH1A3 and no other ALDH isoforms were found to bind to the compound. These results highlight that our strategy of combining detailed structural comparison, structure-based in silico screening followed by biochemical and cell-based analysis can identify and validate selective inhibitors.

Our three-dimensional structure of human ALDH1A3 complexed with MCI-INI-3 provides clarity of the mechanism of inhibition of ALDH1A3 by MCI-INI-3. Structural data reveals that MCI-INI-3 binds to the enzyme active site and overlaps with the retinaldehyde binding pocket, suggestive of a competitive inhibitor. MCI-INI-3 seals off access to the catalytic cysteine with its ester group and interacts with W189, T140, and R139 with its benzodioxole moiety. The selectivity of inhibition of ALDH1A3 by MCI-INI-3 results from the central pyrazolopyrimidine ring of MCI-INI-3 that interacts with N469 and the ester group which interacts with T315. The two structurally equivalent positions in ALDH1A1 are I304 and G458, which cannot form favorable interactions with MCI-INI-3. The structural data of the ALDH1A3/MCI-INI-3 complex explains both the potency and the selectivity of MCI-INI-3 and paves the way for further optimization. As an example, its phenyl moiety could be removed or, on the contrary, functionalized with chemical groups that can engage H128 and N469 to reinforce selectivity and Q304 to improve potency. It should indeed be noted that these three residues are at *Van der Waals* contact distance from MCI-INI-3’s phenyl group and could therefore be exploited for increasing affinity without compromising specificity.

A critical step for the development of a chemical inhibitor of ALDH1A3 is the capacity to inhibit its activity in cells, here focused on GSCs. ALDH1A3 is the predominant ALDH isozyme and plays an important function in RA biosynthesis in MES GSCs. Therefore, we tested the cellular activity of MCI-INI-3 to inhibit ALDH1A3’s function. MCI-INI-3 potently abolished Aldefluor activity in GSCs and in the U87MG glioblastoma cell line, even when the cells were sorted to enrich for Aldefluor-positive cells. Also, when either ROL or RAL were provided in the presence of MCI-INI-3 (15 µM) in U87MG or MES GSC cells, RA, the final product of ALDH1A3, was dramatically decreased. These results are consistent with the loss of Aldefluor reactivity and the impact on RA biosynthesis seen upon ALDH1A3 knockout, suggesting that MCI-INI-3 effectively and selectively inhibited ALDH1A3 activity in cells.

These studies however highlight that the impact of ALDH1A3 loss or inhibition on cancer cell growth is debatable. We developed several different GSC/ALDH1A3-KO cell lines. While each ALDH1A3-KO cell line presented with slightly reduced proliferative capacity, the viability of these KO cells suggests that MES GSCs do not depend on the expression or function of ALDH1A3 for proliferation. This is consistent with the finding that ALDH1A3 was not considered a dependent gene for cell viability when evaluated across a panel of 990 cancer cell lines (https://depmap.org/portal/gene/ALDH1A3)^57^. Further, we found that treatment with either DEAB (a pan-ALDH inhibitor) or MCI-INI-3 leads to a dose-responsive loss of cell viability of both the WT and ALDH1A3-KO cells.

Overall, our results demonstrate that ALDH1A3 plays an important role in regulating the RA signaling pathway in the MES subtype of GSCs. Our in silico screening, combined with biochemical and cell-based analyses, successfully identified MCI-INI-3 as a potent and selective inhibitor of ALDH1A3. Further, structural, and biochemical analyses revealed the mechanism of inhibition and selectivity of MCI-INI-3 against ALDH1A3. MCI-INI-3 inhibited ALDH1A3 activity and altered RA synthesis in MES GSC cells. Future work is needed for the development of a series of small molecule inhibitors of ALDH1A3 based on the structure of MCI-INI-3 and of ALDH1A3 to optimize this new class of agents targeting CSCs with high-ALDH activity. Further development is warranted to characterize the role of ALDH1A3 and RA biosynthesis and its role, if any, in GSC growth and differentiation.

## Materials and methods

### Cells and cell culture conditions

GSCs derived from high-grade glioma samples, the MES subtype (GSC-83, GSC-326) and the PN subtype (GSC-19, GSC-84), were described by us previously^3^. The GSCs were cultured in suspension in GSC growth medium [DMEM-F12 (Cat# 10565, Life Technologies) supplemented with B27 (1:50), heparin (5 mg/mL), basic FGF (bFGF) (20 ng/mL), and EGF (20 ng/mL)] in 100 × 20 mm Petri dishes (Cat# 0875711Z, Fisher Scientific) at 37 °C with 5% CO_2_. Growth medium was changed every 3 or 4 days. U87MG cells (Cat# HTB-14, ATCC) were cultured in EMEM (Cat#11095080, Thermo Fisher Scientific) with 10% FBS(HI), Sodium Pyruvate (1 mM) (Cat#11360-070, Invitrogen), MEM supplemented with Non-Essential Amino acids (0.1 mM) (Cat#11140050, Thermo Fisher Scientific), antibiotic/antimycotic (4 ml) (Cat#15240062, Thermo Fisher Scientific) and Gentamycin (5 µg/ml) (Cat# 9354, Irvine Scientific). Growth medium was changed every 3 or 4 days. 293-FT cells (Cat# R70007, Thermo Fisher Scientific) were cultured in DMEM (Cat# 45000-304, VWR) with 10% FBS(HI), Glutamine (2 mM) (Cat# 25030081, Thermo Fisher Scientific) and antibiotic/antimycotic (4 ml) (Cat# 15240062, Thermo Fisher Scientific). All cells were cultured at 37 °C with 5% CO_2_. Details for each cell line used herein are in Supplementary Table 1.

### Quantitative real-time PCR (qRT-PCR)

The mRNA expression of each ALDH isoform as well as DHRS3 was determined using Taqman Gene Expression Assay probes from Life Technologies:

ALDH1A1: probe ID: Hs00946916_m1

ALDH1A2: probe ID: Hs00180254_m1

ALDH1A3: probe ID: Hs00167476_m1

ALDH1B1: probe ID: Hs04997428_s1

ALDH1L1: probe ID: Hs01003842_m1

ALDH1L2: probe ID: Hs01105342_m1

ALDH2: probe ID: Hs01007998_m1

ALDH3A1: probe ID: Hs00964880_m1

ALDH3A2: probe ID: Hs01116403_m1

ALDH3B1: probe ID: Hs00997594_m1

ALDH3B2: probe ID: Hs02511514_s1

ALDH4A1: probe ID: Hs01013142_m1

ALDH5A1: probe ID: Hs00542449_m1

ALDH6A1: probe ID: Hs00194421_m1

ALDH7A1: probe ID: Hs00609622_m1

ALDH8A1: probe ID: Hs00988965_m1

ALDH9A1: probe ID: Hs00997881_m1

ALDH16A1: probe ID: Hs01035464_m1

ALDH18A1: probe ID: Hs00913261_m1

DHRS3: probe ID: Hs01044021_m1

β-Actin (Cat# 4352935E) was used as an internal control. Each qRT-PCR assay was performed in a 20 μL volume with 4 μL cDNA, 1 μL Taqman probe, 10 μL TaqManFast Universal Master Mix (2x; catalog no. 4367846) and 5 μl of DNase/RNase-free distilled water. The reactions were performed in an ABI StepOnePlus RT-PCR system according to the manufacturer’s protocol. Analysis of mRNA expression was performed as per the instruction of the manufacturer (∆∆CT method). Samples were run in triplicate and the results shown are the mean ± SD of all three analyses. The mRNA level of DHRS3 and each ALDH isoform were then normalized to the expression of human β-Actin (Probe ID: Hs99999903_m1).

### ALDH1A3 knockout by CRISPR/Cas9 in GSC-83, GSC-326, and U87MG cells

Guide RNAs (gRNAs) targeting ALDH1A3 exon 1 or exon 2 were designed using the CRISPR Design Tool^58^, and as described^59^. Each separate gRNA was cloned into pLentiCRISPRv2^60^. Details for each vector developed or used herein are described in Supplementary Table 2. The sequence of each gRNA target sequence and the oligonucleotides used for the vector development are detailed in Supplementary Table 3. The experiment to target the *ALDH1A3* gene was performed as described^60,61^. Briefly, the GSC-83, GSC-326, and U87MG cell lines were transduced by lentivirus prepared from the corresponding gRNA plasmid as we have described previously^62–64^ and described in detail below. Cells were then seeded for selection of single-cell clones and knockout was confirmed by immunoblotting analysis of whole-cell lysates with an ALDH1A3 antibody (Cat# ab129815, Abcam).

### CPOX knockout by CRISPR/Cas9 in U87MG cells

U87MG/CPOX-KO cells were created by transfection of ribonucleoprotein complexes including Cas9 and a mixture of three single-guide RNAs (sgRNAs)^65^ targeting an early exon of the CPOX gene (Synthego). U87MG cells were seeded at a density of 2 × 10^5^ cells per well (six-well plate). After 24 h incubation, the cells were transfected with a mixture of sgRNAs, Cas9 and the CRISPRMAX-Cas9 transfection reagent (Cat# CMAX00008, Thermo Fisher Scientific) in serum-free OptiMEM (Cat# 31985070, Thermo Fisher Scientific). After 48 h, media containing the transfection reagent was replaced with fresh media (Media #2, Supplementary Table 1) and allowed to grow for another 2 days. Validation of gene targeting (knockout, KO) was then confirmed by immunoblot using whole-cell lysates, as compared to a non-targeted control. CPOX antibody (Novus Biologicals, cat# NBP2-59438) was used to confirm the loss of CPOX protein expression after KO and α-Actinin antibody (Cell Signaling, cat# 12413S) was used to confirm expression of the immunoblot loading controls.

### Cellular localization of ALDH1A1, ALDH1A2, or ALDH1A3 in GSC-83 cells

GSC-83 cells were transduced by lentivirus for expression of EGFP-ALDH1A1, EGFP-ALDH1A2, or EGFP-ALDH1A3, as we have described previously^62–64^. Briefly, lentiviral particles were generated by co-transfection of 4 plasmids into 293-FT cells using TransIT-X2 Transfection reagent: the packaging vectors pMD2.g(VSVG), pVSV-REV and pMDLg/pRRE together with the appropriate shuttle vectors, as listed in Supplementary Table 2. Forty-eight hours after transfection, lentivirus-containing supernatant was collected and passed through 0.45 mM filters to isolate the viral particles. Lentiviral transduction was performed as follows: cells (1–2 × 10^5^) were seeded into six-well plates. 24 h later, lentiviral particles were mixed with polybrene (2 µg/ml) and added to the cells. Cells were incubated at 32°C overnight and then medium with lentiviral particles was removed and replaced with fresh medium. Following growth for 48–72 h, cells were seeded into a 4-chamber glass bottom vessel (Cat# 155382, Thermo Fisher Scientific) pre-treated with Cell-Tak™ Cell and Tissue Adhesive (Cat# 354240, Corning) as per the manufacturer’s instructions. EGFP fluorescence was then visualized using a Nikon A1rsi laser scanning confocal microscope (40× oil-immersion objective) to determine the subcellular location of the EGFP-fusion proteins. Nuclei were stained with NucBlue dye.

### Aldefluor assay and separation of cells with high-ALDH activity

ALDH activities of the MES GSC and U87MG cell lines were assayed using the Aldefluor kit per the manufacturer’s instructions (STEMCELL Technologies, Vancouver, BC, Canada). Briefly, 1 × 10^6^ cells were suspended in 1 ml of Aldefluor buffer on ice. Activated Aldefluor (15 μl) substrate was mixed with 1 ml of the cell suspension. Immediately after mixing, 0.5 ml cell suspension was transferred to a tube with 15 μl diethyl amino benzaldehyde (DEAB) and mixed as the negative control. The test sample and negative control were incubated for 30 min at 37 °C and agitated every 5 min. These cells were then centrifuged and resuspended in Aldefluor buffer and kept on ice until analyzed by flow cytometry (BD FACSCanto II). The cells were separated by flow cytometry using a FACSAria III (BD Biosciences, San Jose, CA) based on fluorescence and cell scattering into ALDH^+^ or ALDH^−^ subpopulations. FACS was performed at the Flow Cytometry Core Facility, Mitchel Cancer Institute, University of South Alabama.

### Cell proliferation assay

1500 GSCs (GSC-83 or GSC-326) were seeded in 35 mm dishes with 3 ml growth medium supplemented with DMSO (Control) or MCI-INI-3 (15 µM; Treatment) and incubated for 5 days. The cells were then collected by centrifugation and counted with Trypan Blue assay (3 repeats).

### Dual luciferase assays

The RA reporter firefly luciferase plasmid (pGL3-RARE-luciferase) was purchased from Addgene (Addgene, Plasmid #13458)^66^. Renilla luciferase plasmid is a kind gift from Dr. Ming Tan. After co-transfection of Firefly luciferase and Renilla luciferase plasmids to the control and ALDH1A3-KO cells, the Firefly luciferase and Renilla luciferase activities were determined using the Dual luciferase assay system kit (Promega) per the manufacturer’s instructions. The light intensity was determined with a Synergy H4 Hybrid Multi-Mode Microplate Reader (BioTek) after adding 50 μl of the LAR II reagent (firefly luciferase substrate), and 50 μl of the Stop and Glo reagent (Renilla luciferase substrate), successively. The Firefly luciferase activity was normalized with the Renilla activity (efficiency of transfection) and then normalized to that of the control cells.

### Cloning, expression, and purification of human ALDH1A1 and ALDH1A3

The full-length ALDH1A1 and ALDH1A3 genes were PCR amplified and cloned into the destination vector pDEST17 that leads to the synthesis of an N-term 6xHis tagged protein, using the pENTR/D-TOPO Invitrogen Gateway^®^ recombinant technology and according to the manufacturer’s protocol. The constructed plasmids were then transformed into *Escherichia coli* BL21(DE3) (Novagen) and expressed in a 2xTY medium supplemented with Ampicillin (50 µg/ml). One liter of medium was inoculated with cells from a starter culture and incubated at 37 °C until OD_600_ = 0.6, and subsequently further incubated at 20 °C overnight. Cultures were harvested by centrifugation, and cells resuspended in 40 mL of lysis Buffer A (50 mM Na_2_HPO_4_ pH 7.5, 300 mM NaCl, 1 mM β-mercaptoethanol, 10 mM imidazole) supplemented with 250 U of benzonase nuclease and lysed by sonication on ice. After addition of a protease inhibitor cocktail (100 µL), the lysate was clarified by centrifugation. The soluble fraction was loaded onto a Ni-NTA Superflow 5 mL cartridge (Qiagen) equilibrated with 10 column volumes (CV) of Buffer A and washed with Buffer A supplemented with 50 mM imidazole until the absorbance at 280 nm returned to baseline (15 CV). Then the recombinant protein was eluted with Buffer A supplemented with 250 mM imidazole by a linear gradient in 20 CV. Fractions containing the expected enzyme were pooled and concentrated to 5 ml with Merck Millipore Amicon Ultra-15 10 kDa. The pool was further purified by a gel filtration step on a HiPrep 16/60 Sephacryl 200 High Resolution column equilibrated with a buffer containing 20 mM HEPES pH 7.5, 150 mM KCl, 1 mM β-mercaptoethanol and 0.5 mM EDTA. All the purification steps described above were performed using a BioRad BioLogic DuoFlow FPLC-system. Protein concentration was determined by the Bradford assay, and sample purity was assessed by SDS-PAGE. The protocol allowed the production of pure and active human ALDH1A1 and ALDH1A3 (Supplementary Fig. 4).

### Enzyme inhibition studies

The initial set of potential enzyme inhibitors that were identified by the described structure-based procedure were screened against active recombinant ALDH1A3 and ALDH1A1 by using a previously described continuous spectrometric assay^35^ optimized to suit a Corning^®^ 96-well format plate, that has previously been used to efficiently identify NAD^+^-dependent dehydrogenase inhibitors^67^. For both the ALDH1A3 and ALDH1A1 inhibition test, a 200 μl reaction mixture containing 20 mM Tris-HCl pH 8.0, 1 mM β-mercaptoethanol, 150 mM KCl, 500 μM NAD^+^ and 200 μM acetaldehyde, was set up in a Corning^®^ 96 Well Clear Flat Bottom UV-Transparent Microplate. The reaction was started by the addition of 800 nM pure recombinant enzyme. Change in absorbance at 340 nm (ε_NADH_ = 6220 M^−1^ cm^−1^) was monitored for 30 min in a BioTek ^®^ Synergy 2 Multi-Mode Reader at 25 °C. All the selected potential enzyme inhibitors were dissolved in DMSO and tested at a concentration of 100 μM in triplicate with a final concentration of DMSO in the reaction buffer of 5%. The inhibitory activity of MCI-INI-3 against ALDH1A1 and ALDH1A3 was further investigated to determine enzyme kinetics, the mechanism of inhibition and the inhibitor potency and selectivity. To this end, the enzymatic inhibition assays were performed in a 200 μl total reaction volume in a solution containing 20 mM Tris-HCl, pH 8.0, 1 mM β-mercaptoethanol, 150 mM KCl, 500 μM NAD^+^, 2.6 μM of the pure recombinant enzyme at different acetaldehyde concentrations (1, 5, 10 or 30 mM) and inhibitor concentrations (0, 0.25, 0.5, 5 or 10 μM). For each assay, the reaction mixture was pre-incubated for 1 min in the absence of the enzyme that was subsequently added to initiate the reaction. The kinetic parameters were determined by fitting the measured data to a Michaelis-Menten curve^68^ using SigmaPlot.

### Microarray analysis

Microarray analysis was performed using an Affymetrix GeneAtlas, as per the manufacturer’s instructions and as we have described previously^3^.

### Crystallization and structure determination

Crystals of ALDH1A3 in complex with MCI-INI-3 were obtained by using the vapor-diffusion technique in sitting drop and applying a spare-matrix-based strategy with a crystallization robot (Oryx4, Douglas Instruments). The best crystals were grown by mixing 0.5 μL of the protein solution at a concentration of 7.5 mg/mL, pre-incubated with 1 mM NAD^+^ and 1 mM MCI-INI-3, with an equal volume of a reservoir solution containing 20% PEG-3350, 0.24 M sodium malonate, pH 7.0, and 10 mM TCEP-hydrochloride, equilibrated against 50μL of the reservoir solution at 20 °C for about 30 days. For X-ray data collection, crystals were quickly equilibrated in a solution containing the crystallization buffer and 12.5% glycerol as cryo-protectant and flash frozen at 100 K in liquid nitrogen. Diffraction data up to 2.8 Å resolution were collected at the beamline ID23-EH2 of the European Synchrotron Radiation Facility (ESRF, Grenoble, France). Analysis of the diffraction data set allowed us to assign the crystal to the orthorhombic P2_1_2_1_2_1_ space group with cell dimensions of a = 81.03 Å, b = 158.48 Å, c = 168.65 Å and α = β = γ = 90°, containing four molecules per asymmetric unit with a corresponding solvent content of 65.5%. Diffraction data were processed using the program package XDS^69^ and the CCP4 suite of programs^70^ was used for scaling. The crystal structure determination of the ALDH1A3/MCI-INI-3 complex was carried out by means of the molecular replacement technique using the coordinates of the tetramer of ALDH1A3 as the search model (Protein Data Bank ID code 5FHZ) and the program PHASER^71^. The resulting initial electron density map was of high quality and allowed automatic tracing of the protein chain by the program AUTOBUILDING^72^. The resulting initial model was subjected to iterative cycles of crystallographic refinement with the programs REFMAC5^73^ and PHENIX.REFINE^74^, alternated with manual graphic sessions for model building using the program Coot^75^. Five percent of randomly chosen reflections were excluded from refinement of the structure and used for the Free R-factor calculation^76^. The program ARP/wARP^77^ was used to add solvent molecules. Refinements were continued until convergence to R-factor and free R-factor values of 0.195 and 0.259 respectively, with ideal geometry. Ramachandran statistics show that there are no outliers, and all amino acids are in the favored region. Data collection and refinement statistics are given in Table 1.

### Illustrations

Figures 4, 6, and 7 were generated using the program Pymol (The PyMOL Molecular Graphics System, Version 1.2r3pre, Schrödinger, LLC).

### Cellular thermal stability studies

GSC lysates were snap frozen in liquid nitrogen and the lysate contents were cleared of debris by centrifugation at 25,000 × *g* for 20 min. Thermal stability experiments were carried out at three independent temperatures (45, 50, and 55 °C). Stock solutions of 1 mM MCI-INI-3 were prepared in 100% DMSO. MCI-INI-3 was added to the lysate to a final concentration of 10 μM, protein concentration of 1 mg/ml, resulting in 0.01% DMSO which was matched for control vehicle treatment. The extracts were incubated with compound at room temperature for 1 h and then divided into 50 μL aliquots (*n* = 8) of compound and vehicle treated samples. The samples were heated in parallel at a fixed temperature for 10 min, followed by a 5-min incubation at room temperature. Samples were then centrifuged at 25,000 × *g* for 20 min at 4 °C and the supernatant denatured and digested with trypsin utilizing the FASP method^78,79^. Samples were freeze-dried in a vacuum concentrator and upon desiccation resuspended in 10 μl formic acid in water (0.1%).

Nanoflow LC-MS/MS analysis was performed on an UltiMate3000 nanoLC (Dionex, Sunnydale, CA). Samples (1 μl) were injected via autosampler onto a 25 cm × 75 μM ID reversed phase column packed with 3 μM Reprosil (New Objective, Boston, MA) heated to 50 °C. Peptides were separated and eluted with a gradient from 1 to 28% acetonitrile in 0.1% formic acid over 70 min at 300 nL/min. Samples were injected online into an LTQ-ORBItrap XL mass spectrometer (Thermo Fisher Scientific, Waltham, MA) using a data-dependent top 5 method in positive mode, with spray voltage set at 1.9 kV. Full scan spectra were acquired in the range of m/z 350–1400 at 60,000 resolution using an automatic gain control target of 1 × 10^6^. Tandem mass spectra were acquired in the linear ion trap with a 32.5% normalized collision energy setting and an MSn ion target of 5 × 10^4^. Mass spectrometry data were analyzed on the MaxQuant processing suite (version 1.5.2.8). Spectra were searched against the Uniprot reference database using the MaxQuant built-in peptide identification algorithm, Andromeda. Trypsin was specified as the digestion protease with the possibility of two missed cleavages. Acetylation (protein N-terminus) and oxidation of methionine were set as default variable modifications while carbamidomethylation of cysteine residues was set as a fixed modification. Other database search parameters included 20 ppm and 0.5 Da mass tolerance for precursor and product ions, respectively. Intensities for all peptides were assigned by MaxQuant using full scan mass spectra. Quantified peptides with large variability were filtered using a minimum occupancy filter, median intensity correction, and outlier filter that removes peptides with intensities greater than 3 standard deviations from the mean. The MaxQuant Protein Groups file was then analyzed using Microsoft Excel and statistical significance was established using the Student’s *t* test on log10 intensity values. The GraphPad Prism software was used to generate a volcano scatterplot of the statistical significance versus magnitude of change for each protein group at a given temperature.

### Retinoic acid and metabolite analysis

All-*trans*-retinol (ROL) and all-*trans*-retinaldehyde (RAL) were purchased from Toronto Research Chemicals (Toronto, Canada). One day before treatment, cells were trypsinized, counted and plated into six-well culture plates. On the next day, ROL or RAL was added to the culture medium from ethanol stocks to the final concentration of 10 and 5 µM, respectively. For U87MG cells, the ethanol stock was added directly to the medium. For GSCs, which were cultured without serum, retinoids were first solubilized with equimolar BSA in culture medium, and then dispensed into wells. The inhibitor was added from DMSO stock to a final concentration of 15 µM at the same time as retinoids. U87MG cell lines were incubated with RAL for 5 h, and with ROL—for 24 h. GSC lines were incubated with RAL for 5 h, and with ROL—for 9 h. Each treatment within the experiment was performed in triplicate.

Following the incubation, cells and culture medium were harvested separately, and retinoids were extracted and analyzed by normal phase HPLC as described before^80^ using a Waters 2695 Separation module equipped with a Waters 2996 PDA Detector. The mobile phase was hexane/ethyl acetate/acetic acid (95:4.975:0.025, v/v/v) at a flow rate of 0.7 ml/min. The stationary phase was Waters Spherisorb S3W column (4.6 × 100 mm). Retinoids were quantified by comparing their peak areas to a calibration curve constructed from peak areas of a series of standards. The amount of retinoids produced in each culture well was normalized by the total cellular protein content in the well.

### Statistics and reproducibility

For most analyses, data is shown as the mean ± standard deviation from 2 to 4 independent experiments. Student’s *t* test (unpaired) was used for comparisons between two groups. For multiple comparisons, one-way ANOVA followed by post-hoc Tukey’s multiple comparison test was used. Statistical analysis was performed using GraphPad PRISM v8.

### Reporting summary

Further information on research design is available in the Nature Research Reporting Summary linked to this article.

## Supplementary information

Supplementary Information

Description of Additional Supplementary Files

Supplementary Data 1

Reporting Summary

## Data Availability

Structure deposition: The atomic coordinates and structural factors of human ALDH1A3 in complex with MCI-INI-3 have been deposited with the Protein Data Bank (www.rcsb.org) with the accession code 6TGW (https://www.rcsb.org/structure/6TGW). Proteomic data deposition: The mass spectrometry proteomics data have been deposited to the ProteomeXchange Consortium^81^ via the PRIDE^82^ partner repository with the dataset identifier PXD028125. Microarray data deposition: The microarray data comparing the transcriptome difference between PN and MES GSC cells has been reported previously^3,83^ and can be accessed at: https://www.ncbi.nlm.nih.gov/gds/?term=GSE67089. The microarray data comparing GSC-326(WT) cells to the GSC-326/ALDH1A3-KO cells have been deposited to the GEO database (GSE185463, https://www.ncbi.nlm.nih.gov/geo/query/acc.cgi?acc=GSE185463). Supplementary Data 1: Additional source data used for the preparation of Figs. 1–9. Full-sized uncropped raw images of immunoblots: Summarized in Supplementary Fig. 8. Any remaining information related to the data generated or analyzed in this study is available from the corresponding author upon reasonable request.
