# Supplementary material for: A specific inhibitor of ALDH1A3 regulates retinoic acid biosynthesis in glioma stem cells
Source: Commun Biol. 2021 Dec 21;4:1420. doi: 10.1038/s42003-021-02949-7 (PMC8692581; doi:10.1038/s42003-021-02949-7)
Supplement: Supplementary file 1 — Supplementary Information [file 42003_2021_2949_MOESM1_ESM.pdf]

Supplementary Information

A specific inhibitor of ALDH1A3 regulates retinoic acid biosynthesis in glioma stem cells

Authors
Jianfeng Li[1,2], Silvia Garavaglia[3], Zhaofeng Ye[4,5], Andrea Moretti[3,#], Olga V. Belyaeva[6], Alison Beiser[1,2], Md Ibrahim[1,2], Anna Wilk[1,2], Steve McClellan[1], Alla V. Klyuyeva[6], Kelli R. Goggans[6], Natalia Y. Kedishvili[6], E. Alan Salter[7], Andrzej Wierzbicki[7], Marie E. Migaud[1,2], Steven J. Mullett[8], Nathan A. Yates[8], Carlos J. Camacho[4], Menico Rizzi[3*] and Robert W. Sobol[1,2*]

Affiliations
[1]Mitchell Cancer Institute, University of South Alabama, Mobile, AL 36604, USA
[2]Department of Pharmacology, College of Medicine, University of South Alabama, Mobile, AL 36604, USA
[3]Department of Pharmaceutical Sciences, University of Piemonte Orientale, Largo Donegani 2, 28100 Novara, Italy
[4]Department of Computational and Systems Biology, University of Pittsburgh, Pittsburgh, PA 15261, USA
[5]School of Medicine, Tsinghua University, Beijing, China
[6]Department of Biochemistry and Molecular Genetics, University of Alabama at Birmingham, Schools of Medicine and Dentistry, 720 20th Street South, Kaul 466, Birmingham AL 35294, USA
[7]Department of Chemistry, University of South Alabama, 6040 USA South Drive Mobile, AL 36688, USA
[8]Department of Cell Biology, University of Pittsburgh, Pittsburgh, PA 15261, USA

Supplementary Information includes:

Supplementary Figures 1 – 8

Supplementary Tables 1 – 4

Supplementary Figures

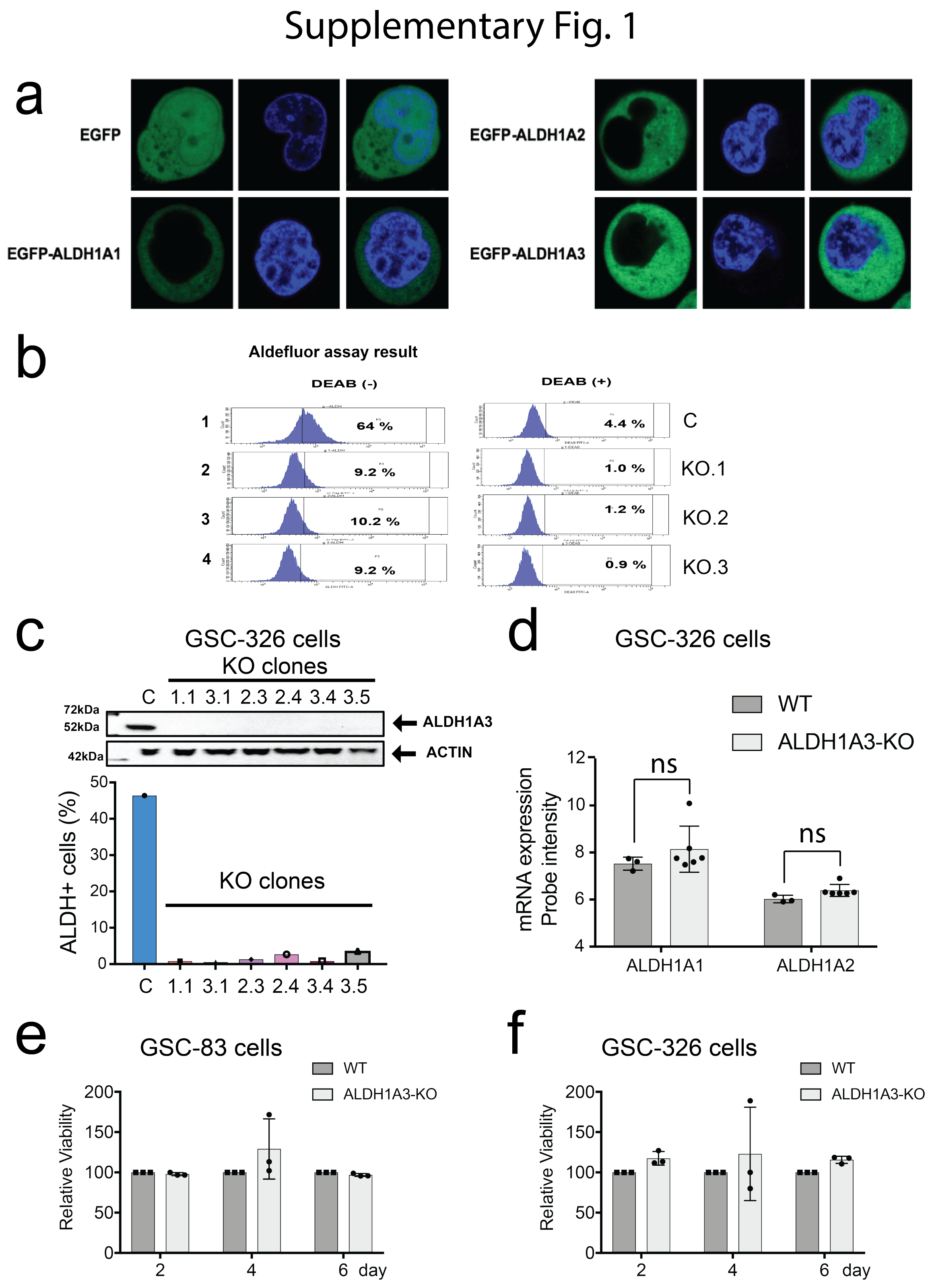

Supplementary Figure 1.

a GSC-83 cells were transduced with lentivirus to express ALDH1A1, ALDH1A2 or ALDH1A3, each as fusion proteins with EGFP. Images show the expression of EGFP, EGFP-ALDH1A1, EGFP-ALDH1A2 or EGFP-ALDH1A3 in GSC-83 cells 48-72 hours post-transduction, highlighting the cytosolic localization of each isoform as per fluorescence confocal microscopy. **b** After acute depletion of ALDH1A3 (three days after lentiviral transduction) in GSC-326 cells with three different guide-RNAs (gRNA), the ALDH activity of those cells were analyzed using the Aldefluor assay with or without DEAB (Pan ALDH inhibitor); 1=GSC-326/CgRNA (control gRNA), 2=GSC-326/ALDH1A3-KO.1 (ALDH1A3-gRNA1), 3=GSC-326/ALDH1A3-KO.2 (ALDH1A3-gRNA2) and 4=GSC-326/ALDH1A3-KO.3 (ALDH1A3-gRNA3). **c** Top-left panel: Immunoblotting analysis confirms the absence of ALDH1A3 protein expression in the GSC-326/ALDH1A3-KO clones (1=GSC-326/CgRNA, 2=GSC-326/ALDH1A3-KO.1.1, 3=GSC-326/ALDH1A3-KO.3.1, 4=GSC-326/ALDH1A3-KO.2.3, 5=GSC-326/ALDH1A3-KO.2.4, 6=GSC-326/ALDH1A3-KO.3.4 and 7=GSC-326/ALDH1A3-KO.3.5). Bottom-left panel: Single-cell clones of GSC-326/ALDH1A3-KO cells were selected, and each clone was analyzed using the Aldefluor assay and quantified as the percentage of $ALDH1^{+}$ cells. **d** The expression of ALDH1A1 and ALDH1A2 was analyzed by microarray analysis in each GSC-326/ALDH1A3-KO clone (n=6, biological repeats) as compared to three WT controls (n=3, biological repeats, p=0.322 for ALDH1A1 and p=0.6603 for ALDH1A2, 2 way ANOVA, ns= not significant). **e** The relative viability of GSC-83 cells vs GSC-83/ALDH1A3-KO (Normalized to WT cells) – viable cells were determined by Trypan Blue exclusion on the 2nd, 4th, and 6th day (n=3, technical replicates). **f** The relative viability of GSC-326 cells vs GSC-326/ALDH1A3-KO (Normalized to WT cells) – viable cells were determined by Trypan Blue exclusion on the 2nd, 4th, and 6th day (n=3, technical replicates).

Supplementary Fig. 2

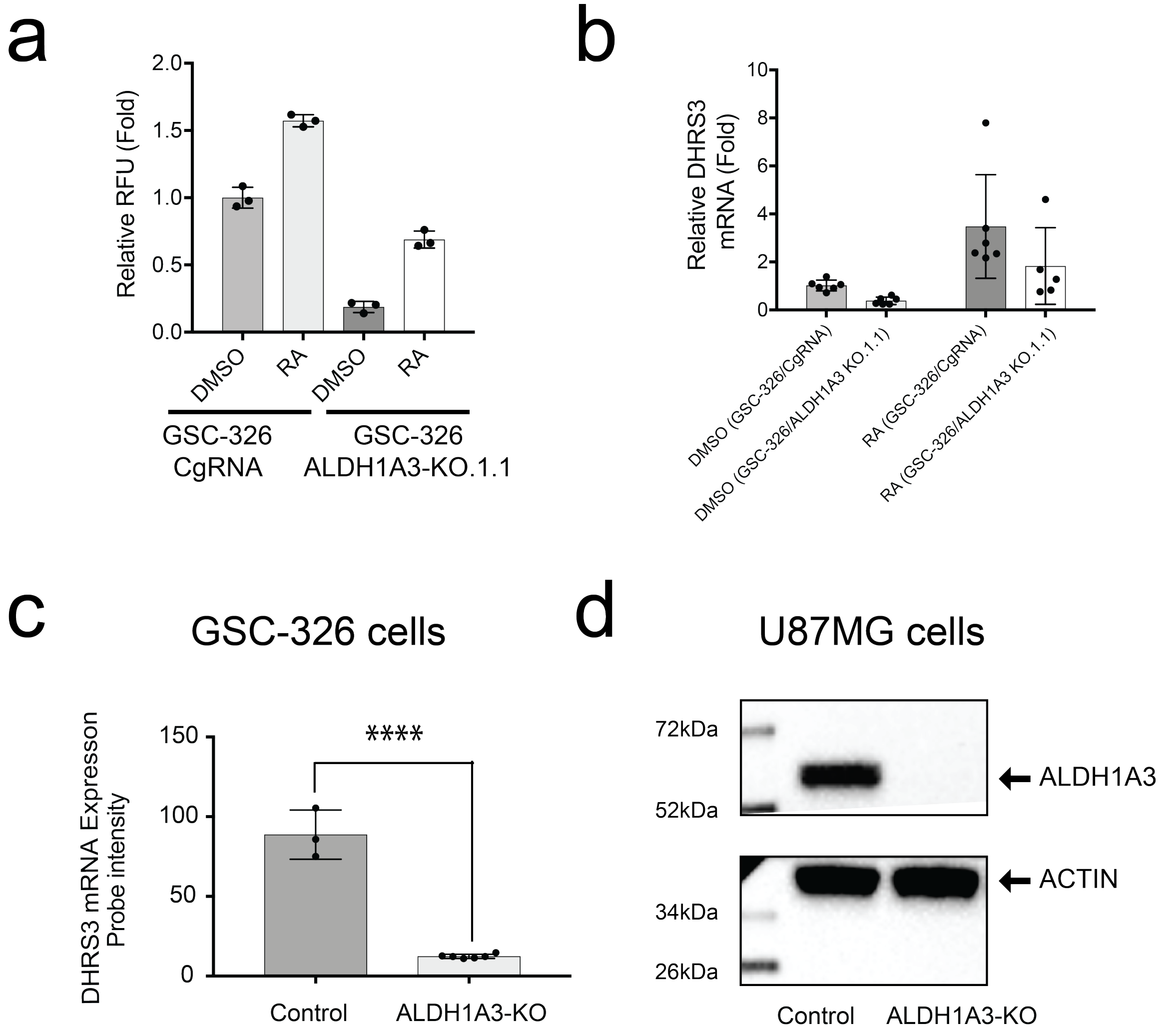

Supplementary Figure 2.

a The GSC-326 control and GSC-326/ALDH1A3-KO.1.1 cells were co-transfected with the reporter vectors for RARE-Luciferase and Renila-Luciferase (the control vector). After transfection (48 hours), DMSO or RA was added, and luminescence was measured and normalized to the GSC-326 control cells (n=3, technical replicates). **b** The GSC-326 control and GSC-83/ALDH1A3-KO.1.1 cells were treated with DMSO or RA for 1 hour and the mRNA level of DHRS3 was analyzed by qRT-PCR, normalized to the GSC-83 control cells (n=6, biological replicates). **c** The expression of DHRS3 was analyzed by microarray analysis in each GSC-326/ALDH1A3-KO clone (n=6, biological repeats) as compared to three WT (GSC-326) controls (n=3, biological repeats, p<0.001, unpaired Student's t-test). **d** Immunoblotting analysis confirms the absence of ALDH1A3 protein in U87MG/ALDH1A3-KO.1 cells.

Supplementary Fig. 3

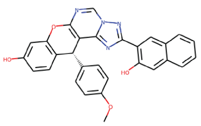

MCI-INI-1

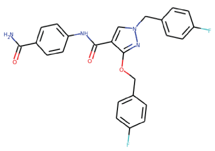

MCI-INI-2

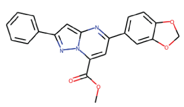

MCI-INI-3

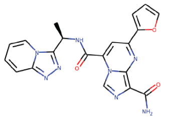

MCI-INI-4

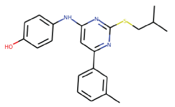

MCI-INI-5

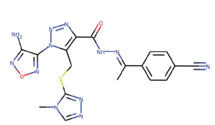

MCI-INI-6

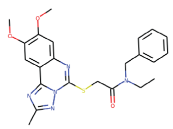

MCI-INI-7

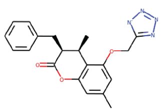

MCI-INI-8

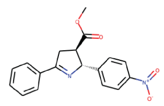

MCI-INI-9

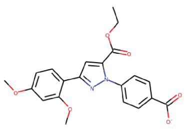

MCI-INI-10

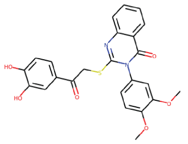

MCI-INI-11

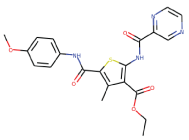

MCI-INI-12

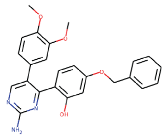

MCI-INI-13

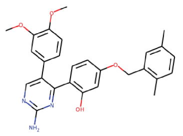

MCI-INI-14

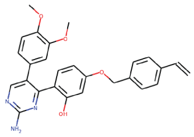

MCI-INI-15

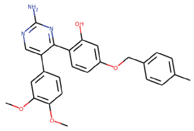

MCI-INI-16

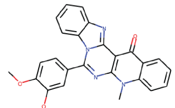

MCI-INI-18

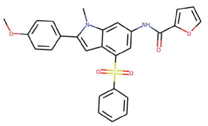

MCI-INI-19

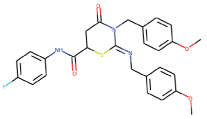

MCI-INI-20

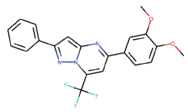

MCI-INI-21

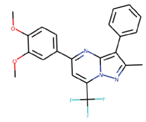

MCI-INI-22

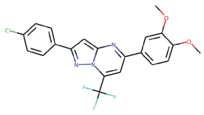

MCI-INI-23

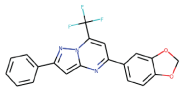

MCI-INI-24

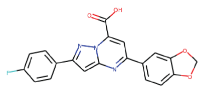

MCI-INI-25

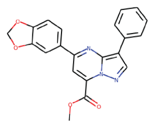

MCI-INI-26

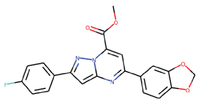

MCI-INI-27

Supplementary Figure 3.

Structures of the compounds identified and analyzed (see also **Supplementary Table 4**).

Supplementary Fig. 4

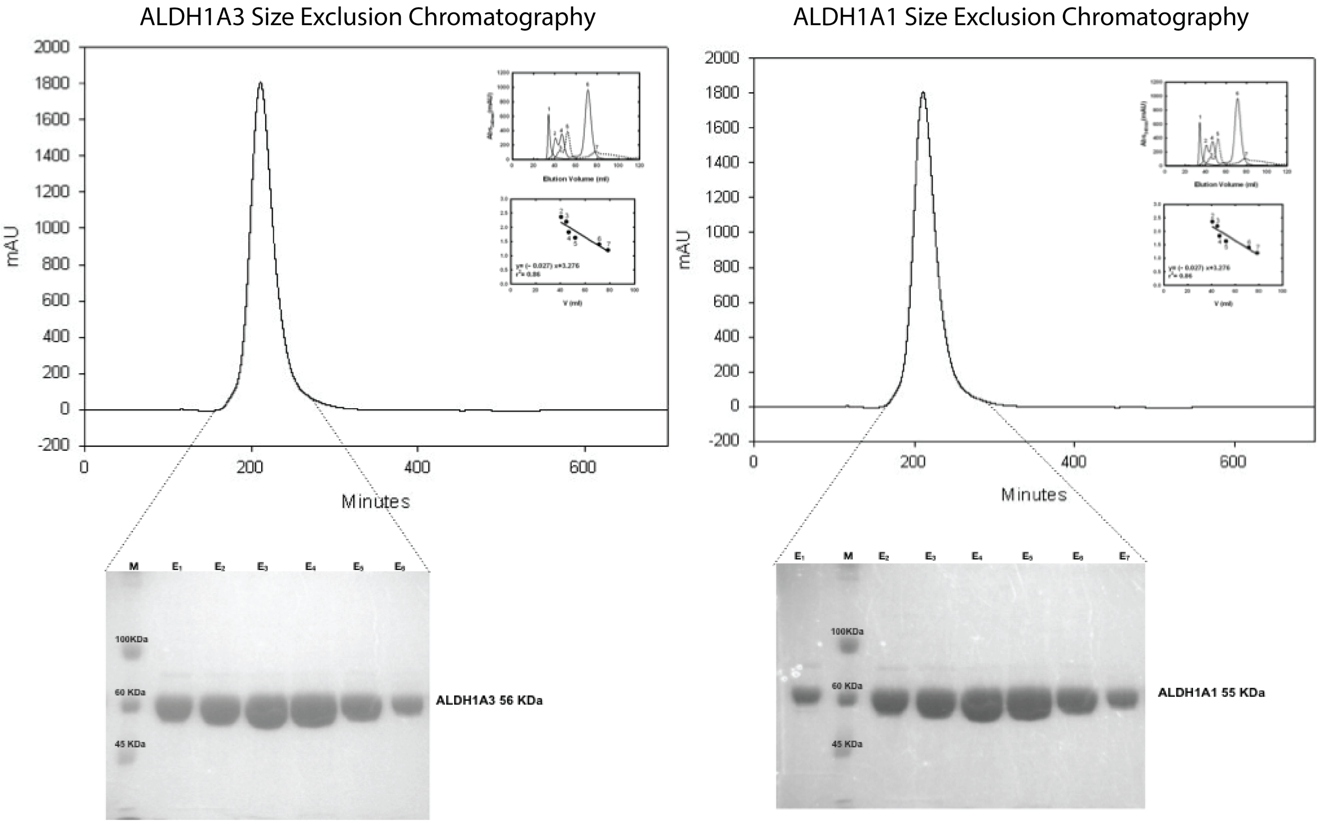

Supplementary Figure 4.

Size exclusion chromatograms on HiPrep 16/60 Sephacryl 200 High Resolution columns using a BioRad BioLogic DuoFlow FPLC-systems for ALDH1A3 (left) and ALDHA1 (right). The sample purity was assessed by 12% SDS-PAGE and is reported in the lower part of the figure for the two isozymes.

Supplementary Fig. 5

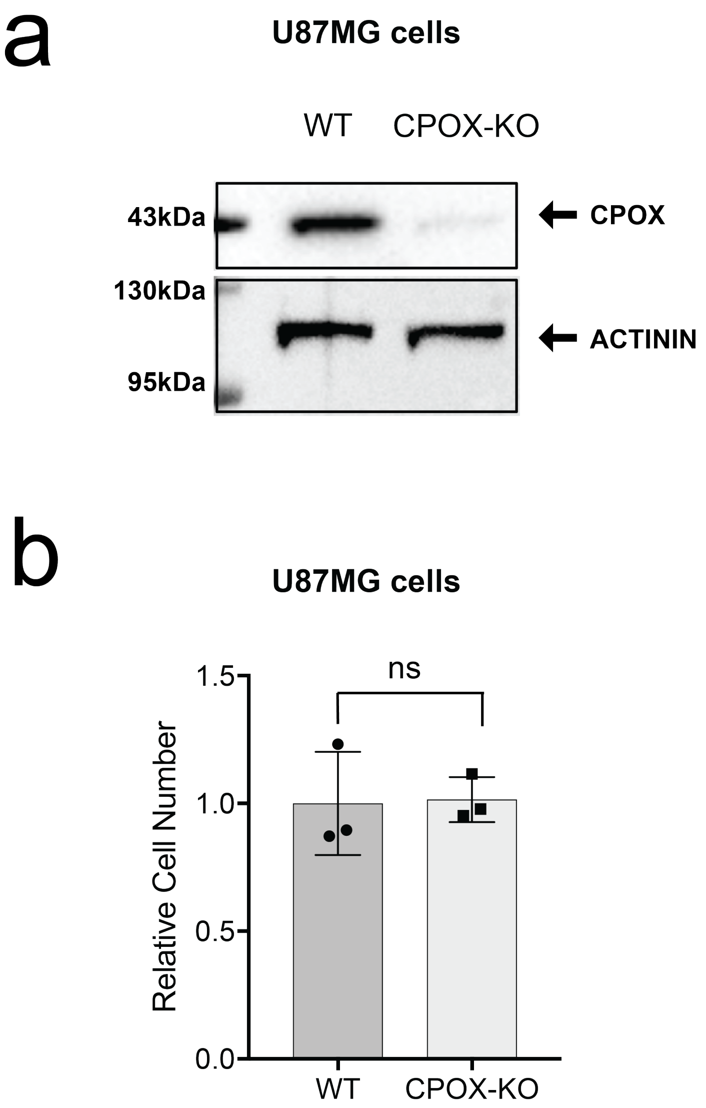

Supplementary Figure 5.

a Immunoblotting analysis of CPOX in U87MG (WT) and U87MG/CPOX-KO cells. Actinin was used as the loading control. **b** U87MG (WT) or U87MG/CPOX-KO cells (0.1 x 10^6) were seeded in a 100mm dish and incubated for 7 days. Viable cells were counted, and the relative cell number of cells was plotted, normalized to the U87MG (WT) cells (n=3, biological repeats, p=0.910, an unpaired Student's t-test, ns= not significant).

Supplementary Fig. 6

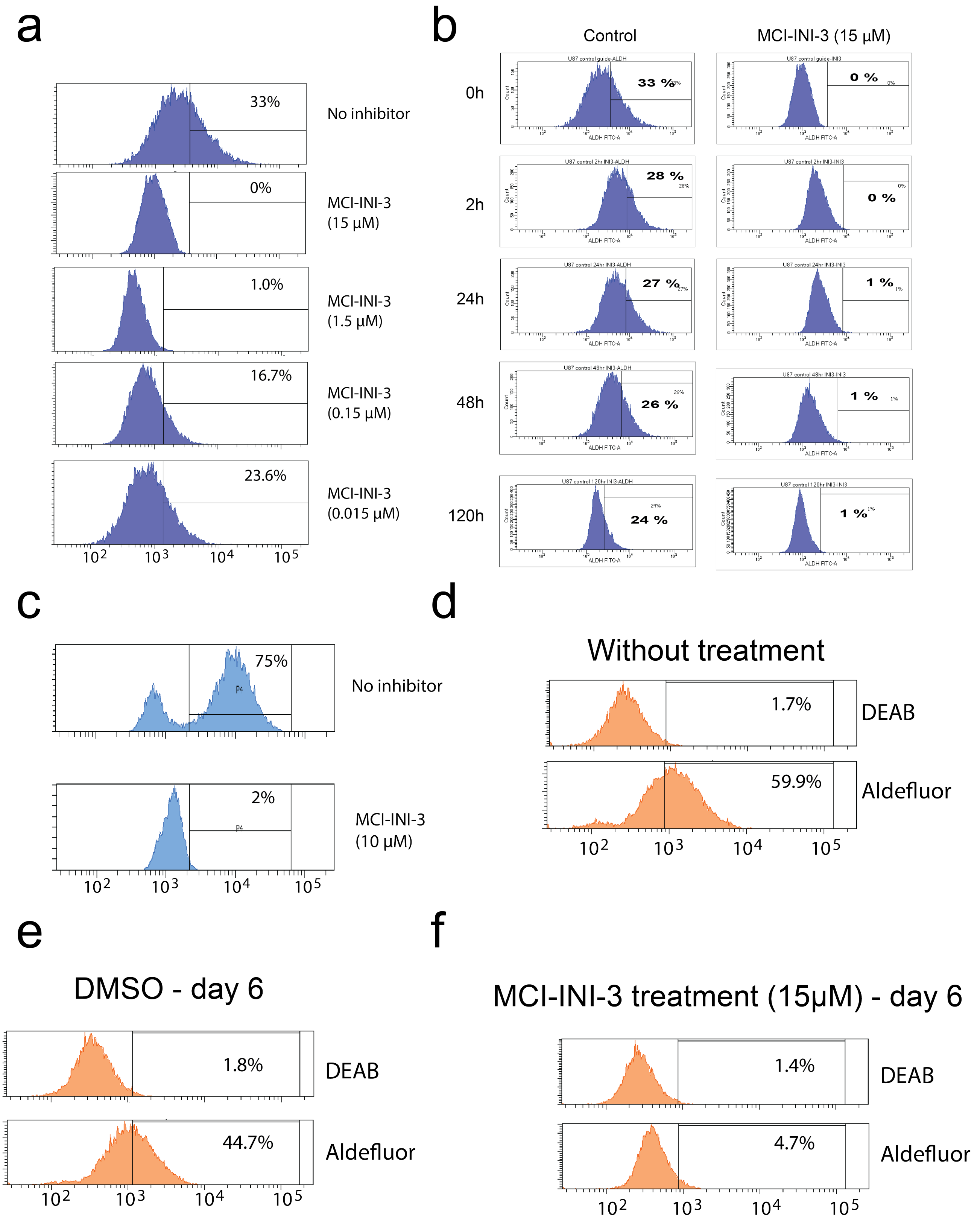

Supplementary Figure 6.

a Regulation of ALDH activity in U87MG cells treated with varying concentrations of MCI-INI-3 as measured with the Aldefluor assay. **b** Inhibition of ALDH1A3 activity by MCI-INI-3: U87MG cells were treated with MCI-INI-3 (15μM) in culture medium. The cells were dissociated and the ALDH activity was measured by the Aldefluor assay at different time points. **c** U87MG cells were sorted using Aldefluor-FACS to enrich the ALDH positive population to 75%. The enriched cells were then treated with MCI-INI-3 (10 μM) for 15 minutes. The ALDH activity was assayed by the Aldefluor assay. **d** The ALDH activity of the GSC-326 cells were analyzed using the Aldefluor assay without treatment. DEAB was used as a negative control. **e** After a 6-day treatment DMSO, the ALDH activity of the GSC-326 cells was analyzed using the Aldefluor assay. DEAB was used as a negative control. **f** After a 6-day treatment of MCI-INI-3 (15 μM), the ALDH activity of the GSC-326 cells were analyzed using the Aldefluor assay. DEAB was used as a negative control.

Supplementary Fig. 7

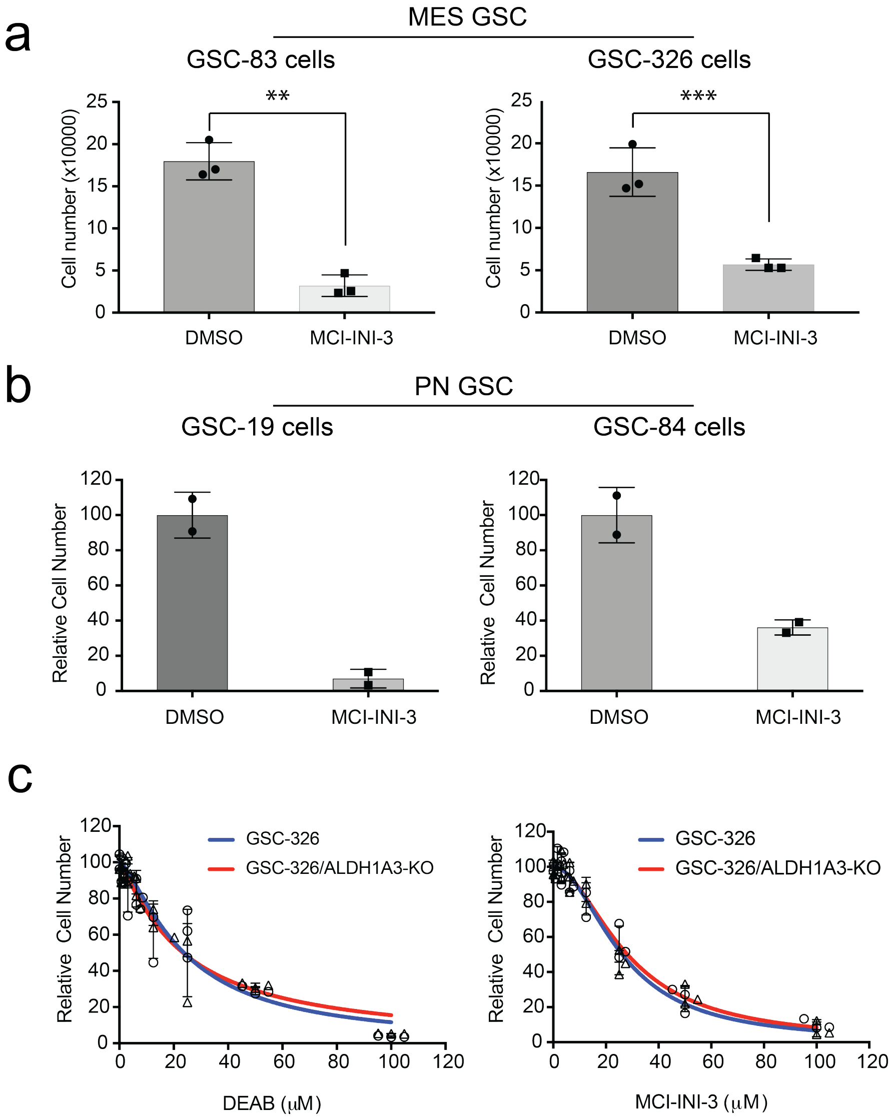

Supplementary Figure 7.

a The MES GSC-83 and GSC-326 cells were treated with MCI-INI-3 (15μM) for 5 days. Viable cells were counted and plotted (GSC-83: n=3 biological repeats, p=0.0006, an unpaired Student's t-test; GSC-326: n=3 biological repeats, p=0.003, an unpaired Student's t-test). **b** The PN GSC-19 and GSC-84 cells were treated with MCI-INI-3 (15μM) for 5 days. Viable cells were counted and plotted (n=2 biological repeats). **c** Dose response analysis of DEAB (left) or MCI-INI-3 (right) treatment of GSC-326 and GSC-326/ALDH1A3-KO cells. Cells were incubated in the absence or presence of the compound for 6 days. Viable cells were counted and plotted. IC_{50} values were calculated using GraphPad Prism 8 (n=3 technique repeats). Statistical analysis was performed using GraphPad Prism 8 (ns= not significant, *<0.05, **<0.01, ***<0.001 or ****<0.0001).

Supplementary Fig. 8 (page 1)

a Fig. 2b uncropped images

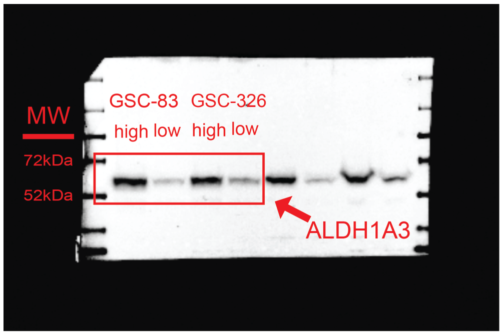

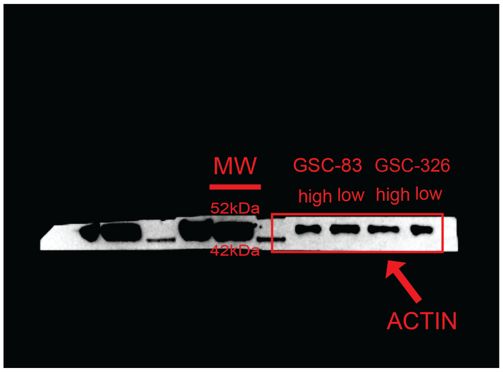

b Fig. 2c uncropped images

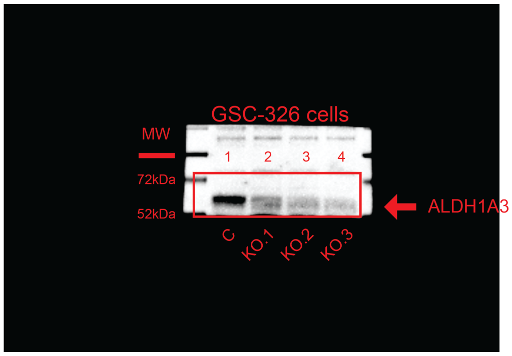

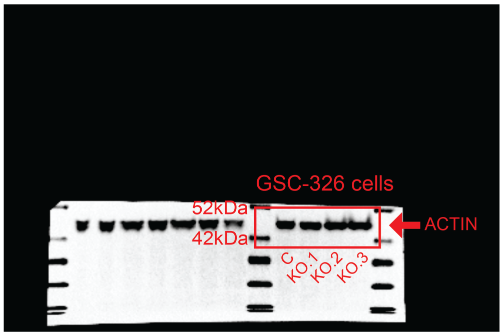

c Fig. 2e uncropped images

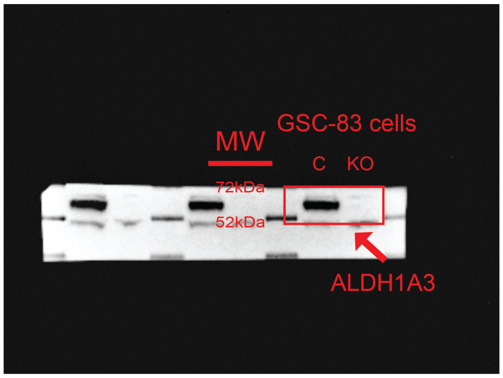

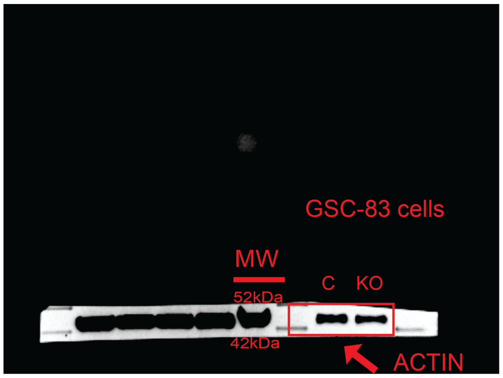

d Fig. 2f uncropped images

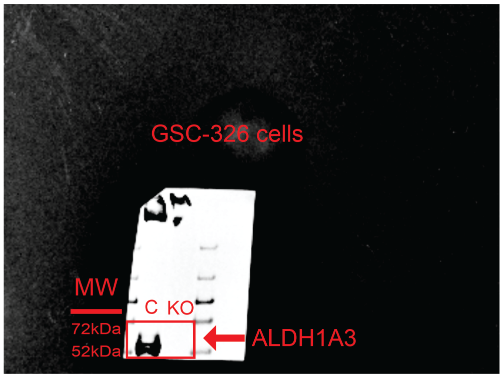

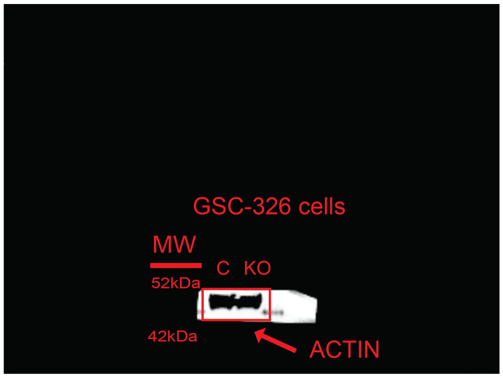

Supplementary Fig. 8 (page 2)

e Supplementary Fig. 1c uncropped images

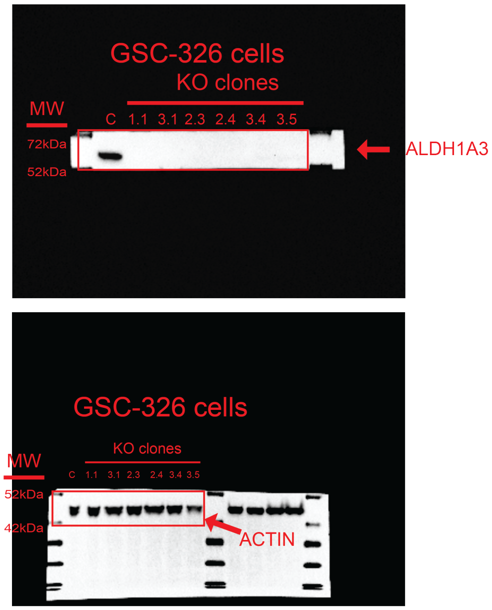

f Supplementary Fig. 2c uncropped images

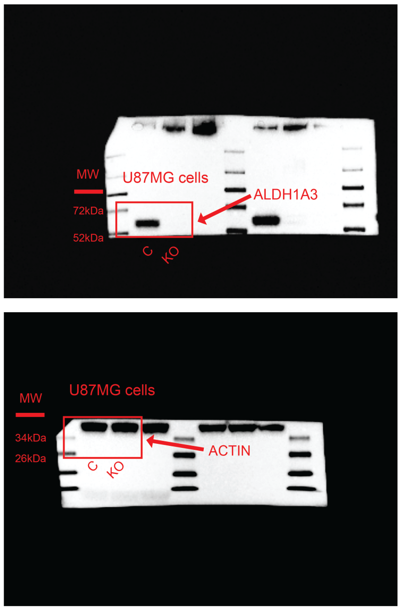

g Supplementary Fig. 5b uncropped images

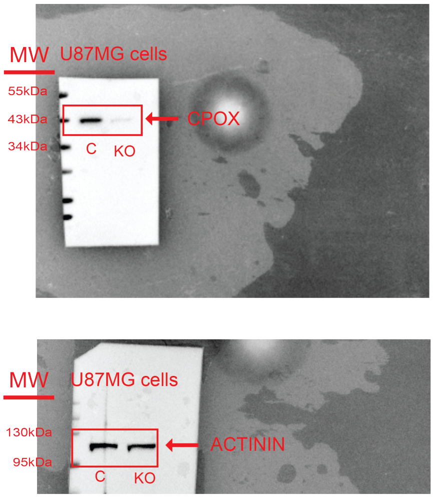

Supplementary Figure 8. Full scanned, uncropped immunoblot images.

a Uncropped immunoblots from Fig. 2b. **b** Uncropped immunoblots from Fig. 2c. **c** Uncropped immunoblots from Fig. 2e. **d** Uncropped immunoblots from Fig. 2f. **e** Uncropped immunoblots from Supplementary Fig. 1c. **f** Uncropped immunoblots from Supplementary Fig. 2c. **g** Uncropped immunoblots from Supplementary Fig. 5b.

Supplementary Tables

Supplementary Table 1. Human cell lines developed for and/or used in this study

Cell line name	Cell line description	Growth media*
GSC-83	Glioma stem cells, Mesenchymal (MES) subtype	Media #1
GSC-326	Glioma stem cells, Mesenchymal (MES) subtype	Media #1
GSC-19	Glioma stem cells, Proneural (PN) subtype	Media #1
GSC-84	Glioma stem cells, Proneural (PN) subtype	Media #1
GSC-83/CgRNA	GSC-83 cells expressing Cas9 and a control gRNA (pool)	Media #1
GSC-83/ALDH1A3-KO.1	GSC-83 cells expressing Cas9 and ALDH1A3 gRNA1 (pool)	Media #1
GSC-83/ALDH1A3-KO.1.2	GSC-83 cells expressing Cas9 and ALDH1A3 gRNA1, clone 2	Media #1
GSC-326/CgRNA	GSC-326 cells expressing Cas9 and a control gRNA (pool)	Media #1
GSC-326/ALDH1A3-KO.1	GSC-326 cells expressing Cas9 and ALDH1A3 gRNA1 (pool)	Media #1
GSC-326/ALDH1A3-KO.2	GSC-326 cells expressing Cas9 and ALDH1A3 gRNA2 (pool)	Media #1
GSC-326/ALDH1A3-KO.3	GSC-326 cells expressing Cas9 and ALDH1A3 gRNA3 (pool)	Media #1
GSC-326/ALDH1A3-KO.1.1	GSC-326 cells expressing Cas9 and ALDH1A3 gRNA1, clone 1	Media #1
GSC-326/ALDH1A3-KO.3.1	GSC-326 cells expressing Cas9 and ALDH1A3 gRNA3, clone 1	Media #1
GSC-326/ALDH1A3-KO.2.3	GSC-326 cells expressing Cas9 and ALDH1A3 gRNA2, clone 3	Media #1
GSC-326/ALDH1A3-KO.2.4	GSC-326 cells expressing Cas9 and ALDH1A3 gRNA2, clone 4	Media #1
GSC-326/ALDH1A3-KO.3.4	GSC-326 cells expressing Cas9 and ALDH1A3 gRNA3, clone 4	Media #1
GSC-326/ALDH1A3-KO.3.5	GSC-326 cells expressing Cas9 and ALDH1A3 gRNA3, clone 5	Media #1
U87MG	Glioblastoma cell line	Media #2
U87MG/CgRNA	U87MG cells expressing Cas9 and a control gRNA (pool)	Media #3
U87MG/ALDH1A3-KO.1	U87MG cells expressing Cas9 and ALDH1A3 gRNA1 (pool)	Media #3
U87MG/CPOX-KO	U87MG cells modified by CRISPR/cas9 at the CPOX locus	Media #2
293FT	Human embryonal kidney cells transformed with the SV40 large T antigen	Media #4

*Media #1 (GSC growth Medium): DMEM-F12 (Cat# 10565, Life Technologies) supplemented with B27 (1:50), heparin (5 mg/mL), basic FGF (bFGF) (20 ng/mL), and EGF (20 ng/mL) and 1x antibiotic/antimycotic (Life Technologies; #15240-096).

*Media #2: EMEM with 10% FBS (heat inactivated), 1mM Sodium Pyruvate, 10mM MEM non-essential amino acids, 1x antibiotic/antimycotic (Life Technologies; #15240-096) and 5µg/mL Gentamycin.

*Media #3: Media #2 supplemented with Puromycin (1.0µg/ml).

*Media #4" DMEM (Cat#45000-304, VWR) with 10% FBS(HI), Glutamine (2mM) (Cat#25030081, Thermo Fisher Scientific) and antibiotic/antimycotic (4ml) (Cat#15240062, Thermo Fisher Scientific).

Supplementary Table 2. Vectors developed for and used in this study

Plasmid name	Insert description
pLenti-PGK-GFPc3-Neo	GFP
pLenti-PGK-EGFP-ALDH1A1-Neo A	EGFP-ALDH1A1
pLenti-PGK-EGFP-ALDH1A2-Neo (clone 12)	EGFP-ALDH1A2
pLenti-PGK-EGFP-ALDH1A3-Neo (clone 4)	EGFP-ALDH1A3
pLenti-CRISPR-v2	Cas9
pLenti-v2-ALDH1A3gRNA1	Cas9 + ALDH1A3/gRNA1
pLenti-v2-ALDH1A3gRNA2	Cas9 + ALDH1A3/gRNA2
pLenti-v2-ALDH1A3gRNA3	Cas9 + ALDH1A3/gRNA3
pDEST17-ALDH1A1	6XHISTag-hALDH1A1
pDEST17-ALDH1A3	6XHISTag-hALDH1A3

Supplementary Table 3. Oligonucleotides used in this study

Oligonucleotide name	Sequence 5' – 3'
gRNA target – g1	GTCCGGCTGCCCGTTTTCCA
ALDH1A3-gR-1-F	CACCGGTCCGGCTGCCCGTTTTCCA
ALDH1A3-gR-1-R	AAACTGGAAAACGGGCAGCCGGACC
gRNA target – g2	AGCCATGGCCACCGCTAACG
ALDH1A3-gR-2-F	CACCGAGCCATGGCCACCGCTAACG
ALDH1A3-gR-2-R	AAACCGTTAGCGGTGGCCATGGCTC
gRNA target – g3	TGAACTTGACCTCCAGGTTG
ALDH1A3-gR-3-F	CACCGTGAACTTGACCTCCAGGTTG
ALDH1A3-gR-3-R	AAACCAACCTGGAGGTCAAGTTCAC

Supplementary Table 4. ALDH1A3 inhibitors evaluated in this study

Cmp. Names	Inhibition Summary	ZINC ID	Affinity	Mol Wt	MolPort ID	Formula
MCI-INI-A1	No inhibition	ZINC09584070	-10.45588	488.4935	MolPort-000-800-188	$C_{29}H_{20}N_4O_4$
MCI-INI-A2	No inhibition	ZINC09101414	-10.21557	462.4481	MolPort-003-120-358	$C_{25}H_{20}F_2N_4O_3$
MCI-INI-A3	Strong inhibition	ZINC20606903	-9.93482	373.3615	MolPort-002-780-992	$C_{21}H_{15}N_3O_4$
MCI-INI-A4	No inhibition	ZINC40062050	-9.34422	416.3928	MolPort-009-264-363	$C_{20}H_{16}N_8O_3$
MCI-INI-A5	No inhibition	ZINC11907658	-8.87209	401.953	MolPort-008-825-710	$C_{21}H_{24}ClN_3OS$
MCI-INI-A6	No inhibition	ZINC09307231	-8.73627	464.464	MolPort-002-626-578	$C_{18}H_{16}N_{12}O_2S$
MCI-INI-A7	No inhibition	ZINC09838546	-8.38599	451.541	MolPort-007-630-896	$C_{23}H_{25}N_5O_3S$
MCI-INI-A8	No inhibition	ZINC39980280	-8.11449	362.3819	MolPort-005-977-395	$C_{20}H_{18}N_4O_3$
MCI-INI-A9	Weak inhibition	ZINC01096684	-8.05425	324.3306	MolPort-003-876-986	$C_{18}H_{16}N_2O_4$
MCI-INI-A10	No inhibition	ZINC02622163	-7.2393	396.3933	MolPort-004-035-340	$C_{21}H_{20}N_2O_6$
MCI-INI-A11	Weak inhibition	ZINC28849234	-6.6453	464.49	MolPort-009-245-021	$C_{24}H_{20}N_2O_6S$
MCI-INI-A12	No inhibition	ZINC19631637	-5.68021	440.472	MolPort-008-296-072	$C_{21}H_{20}N_4O_5S$
MCI-INI-A13	No inhibition	ZINC02343889	-5.3782	429.4678	MolPort-002-630-781	$C_{25}H_{23}N_3O_4$
MCI-INI-A14	Weak inhibition	ZINC11866288	-5.33404	457.521	MolPort-002-658-515	$C_{27}H_{27}N_3O_4$
MCI-INI-A15	No inhibition	ZINC09430465	-5.01415	455.5051	MolPort-000-821-580	$C_{27}H_{25}N_3O_4$
MCI-INI-A16	No inhibition	ZINC02350903	-4.95129	443.4944	MolPort-002-645-752	$C_{26}H_{25}N_3O_4$
MCI-INI-A18	No inhibition	ZINC09327945	-10.432	436.471	MolPort-002-649-615	$C_{26}H_{20}N_4O_3$
MCI-INI-A19	No inhibition	ZINC09610809	-8.1448	486.54	MolPort-007-646-010	$C_{27}H_{22}N_2O_5S$
MCI-INI-A20	No inhibition	ZINC15320296	-5.4747	507.58	MolPort-002-728-279	$C_{27}H_{26}FN_3O_4S$
MCI-INI-A21	No inhibition	ZINC01040254	n/d	399.373	MolPort-001-809-311	$C_{21}H_{16}F_3N_3O_2$
MCI-INI-A22	No inhibition	ZINC02874408	n/d	413.4	MolPort-002-288-128	$C_{22}H_{18}F_3N_3O_2$
MCI-INI-A23	No inhibition	ZINC01040257	n/d	433.82	MolPort-002-909-242	$C_{21}H_{15}ClF_3N_3O_2$
MCI-INI-A24	No inhibition	ZINC01040220	n/d	383.33	MolPort-001-809-264	$C_{20}H_{12}F_3N_3O_2$
MCI-INI-A25	Weak inhibition	ZINC36615757	n/d	377.331	MolPort-002-781-067	$C_{20}H_{12}FN_3O_4$
MCI-INI-A26	No inhibition	ZINC13645363	n/d	373.368	MolPort-002-780-848	$C_{21}H_{15}N_3O_4$
MCI-INI-A27	No inhibition	ZINC36615747	n/d	391.358	MolPort-002-781-021	$C_{21}H_{14}FN_3O_4$

*Used for co-crystal structure

Cmp. Names	Simplified Molecular Input Line Entry System (SMILES)
MCI-INI-A1	[H]OC1=CC2=C(C=C1)[C@@H](C1=CC=C(OC)C=C1)C1=C(O2)N=CN2N=C(N=C12)C1=C(O[H])C=C2C=CC=CC2=C1
MCI-INI-A2	[H]N([H])C(=O)C1=CC=C(C=C1)N([H])C(=O)C1=CN(CC2=CC=C(F)C=C2)N=C1OCC1=CC=C(F)C=C1
MCI-INI-A3	COC(=O)c1cc(nc2n1nc(c2)c3ccccc3)c4ccc5c(c4)OCO5
MCI-INI-A4	[H]N([H])C(=O)C1=C2N=C(C=C(N2C=N1)C(=O)N([H])[C@H](C)C1=NN=C2C=CC=CN12)C1=CC=CO1
MCI-INI-A5	[H]OC1=CC=C(C=C1)N([H])C1=CC(=NC(SCC(C)C)=N1)C1=CC=CC(C)=C1
MCI-INI-A6	[H]N([H])C1=NON=C1N1N=NC(C(=O)N([H])\N=C(/C)C2=CC=C(C=C2)C#N)=C1CSC1=NN=CN1C
MCI-INI-A7	CCN(Cc1ccccc1)C(=O)CSc2nc3cc(c(cc3c4n2nc(n4)C)OC)OC
MCI-INI-A8	Cc1cc2c(c(c1)OCc3[n-]nnn3)[C@@H]([C@@H](C(=O)O2)Cc4ccccc4)C
MCI-INI-A9	COC(=O)[C@@H]1CC(=N[C@H]1c2ccc(cc2)[N+](=O)[O-])c3ccccc3
MCI-INI-A10	CCOC(=O)c1cc(nn1c2ccc(cc2)C(=O)[O-])c3ccc(cc3OC)OC
MCI-INI-A11	COc1ccc(cc1OC)n2c(=O)c3ccccc3nc2SCC(=O)c4ccc(c(c4)O)O
MCI-INI-A12	[H]N(C(=O)C1=NC=CN=C1)C1=C(C(=O)OCC)C(C)=C(S1)C(=O)N([H])C1=CC=C(OC)C=C1
MCI-INI-A13	[H]OC1=C(C=CC(OCC2=CC=CC=C2)=C1)C1=C(C=NC(=N1)N([H])[H])C1=CC(OC)=C(OC)C=C1
MCI-INI-A14	[H]OC1=C(C=CC(OCC2=C(C)C=CC(C)=C2)=C1)C1=NC(=NC=C1C1=CC(OC)=C(OC)C=C1)N([H])[H]
MCI-INI-A15	[H]OC1=C(C=CC(OCC2=CC=C(C=C)C=C2)=C1)C1=C(C=NC(=N1)N([H])[H])C1=CC(OC)=C(OC)C=C1
MCI-INI-A16	[H]OC1=C(C=CC(OCC2=CC=C(C)C=C2)=C1)C1=NC(=NC=C1C1=CC(OC)=C(OC)C=C1)N([H])[H]
MCI-INI-A18	COc1ccc(cc1OC)-c1nc2n(C)c3ccccc3c(=O)c2c2nc3ccccc3n12
MCI-INI-A19	COc1ccc(cc1)-c1cc2c(cc(NC(=O)c3ccco3)cc2n1C)S(=O)(=O)c1ccccc1
MCI-INI-A20	COc1ccc(C\N=C2/SC(CC(=O)N2Cc2ccc(OC)cc2)C(=O)Nc2ccc(F)cc2)cc1
MCI-INI-A21	COc1ccc(cc1OC)-c1cc(n2nc(cc2n1)-c1ccccc1)C(F)(F)F
MCI-INI-A22	COc1ccc(cc1OC)-c1cc(n2nc(C)c(-c3ccccc3)c2n1)C(F)(F)F
MCI-INI-A23	COc1ccc(cc1OC)-c1cc(n2nc(cc2n1)-c1ccc(Cl)cc1)C(F)(F)F
MCI-INI-A24	FC(F)(F)c1cc(nc2cc(nn12)-c1ccccc1)-c1ccc2OCOc2c1
MCI-INI-A25	OC(=O)c1cc(nc2cc(nn12)-c1ccc(F)cc1)-c1ccc2OCOc2c1
MCI-INI-A26	COC(=O)c1cc(nc2c(cnn12)-c1ccccc1)-c1ccc2OCOc2c1
MCI-INI-A27	COC(=O)c1cc(nc2cc(nn12)-c1ccc(F)cc1)-c1ccc2OCOc2c1

*Used for co-crystal structure
