# Supplementary material for: A specific inhibitor of ALDH1A3 regulates retinoic acid biosynthesis in glioma stem cells
Source: Commun Biol. 2021 Dec 21;4:1420. doi: 10.1038/s42003-021-02949-7 (PMC8692581; doi:10.1038/s42003-021-02949-7)
Supplement: Supplementary file 2 — Description of Additional Supplementary Files [file 42003_2021_2949_MOESM2_ESM.pdf]

Description of Additional Supplementary Files

File name: Supplementary Data 1
Description: Source data underlying the main figure panels
