# Supplementary material for: A specific inhibitor of ALDH1A3 regulates retinoic acid biosynthesis in glioma stem cells
Source: Commun Biol. 2021 Dec 21;4:1420. doi: 10.1038/s42003-021-02949-7 (PMC8692581; doi:10.1038/s42003-021-02949-7)
Supplement: Supplementary file 4 — Reporting Summary [file 42003_2021_2949_MOESM4_ESM.pdf]

nature research

Corresponding author(s): Menico Rizzi
Robert W. Sobol
COMMSBIO-20-0294-T

Last updated by author(s): Nov 11, 2021

Reporting Summary

Nature Research wishes to improve the reproducibility of the work that we publish. This form provides structure for consistency and transparency in reporting. For further information on Nature Research policies, see our Editorial Policies and the Editorial Policy Checklist.

Statistics

For all statistical analyses, confirm that the following items are present in the figure legend, table legend, main text, or Methods section.

n/a	Confirmed	
☐	☒	The exact sample size (n) for each experimental group/condition, given as a discrete number and unit of measurement
☐	☒	A statement on whether measurements were taken from distinct samples or whether the same sample was measured repeatedly
☐	☒	The statistical test(s) used AND whether they are one- or two-sided<br>*Only common tests should be described solely by name; describe more complex techniques in the Methods section.*
☒	☐	A description of all covariates tested
☒	☐	A description of any assumptions or corrections, such as tests of normality and adjustment for multiple comparisons
☐	☒	A full description of the statistical parameters including central tendency (e.g. means) or other basic estimates (e.g. regression coefficient) AND variation (e.g. standard deviation) or associated estimates of uncertainty (e.g. confidence intervals)
☒	☐	For null hypothesis testing, the test statistic (e.g. F, t, r) with confidence intervals, effect sizes, degrees of freedom and P value noted<br>*Give P values as exact values whenever suitable.*
☒	☐	For Bayesian analysis, information on the choice of priors and Markov chain Monte Carlo settings
☒	☐	For hierarchical and complex designs, identification of the appropriate level for tests and full reporting of outcomes
☒	☐	Estimates of effect sizes (e.g. Cohen's d, Pearson's r), indicating how they were calculated

Our web collection on statistics for biologists contains articles on many of the points above.

Software and code

Policy information about availability of computer code

Data collection	Crystallization and structure determination: Diffraction data were processed using the program package XDS (Kabsch 2010) and the CCP4 suite of programs (Collaborative Computational Project 1994) was used for scaling.<br>Enzyme inhibition studies: The kinetic parameters were determined by fitting the measured data to a Michaelis-Menten curve (Michaelis and Menten 1913) using SigmaPlot.<br>Cellular thermal stability studies: The MaxQuant Protein Groups file was then analyzed using Microsoft Excel and statistical significance was established using the Student's t test on log10 intensity values. The GraphPad Prism software was used to generate a volcano scatterplot of the statistical significance versus magnitude of change for each protein group at a given temperature.<br>Statistical Analysis: For most analyses, data is shown as the mean +/- standard deviation from 2-4 independent experiments. Student's t-test (unpaired) was used for comparisons between two groups. For multiple comparisons, one-way ANOVA followed by post-hoc Tukey's multiple comparison test was used. Statistical analysis was performed using GraphPad PRISM v8.
Data analysis	See above. All detailed in the Methods.

For manuscripts utilizing custom algorithms or software that are central to the research but not yet described in published literature, software must be made available to editors and reviewers. We strongly encourage code deposition in a community repository (e.g. GitHub). See the Nature Research guidelines for submitting code & software for further information.

Data

Policy information about availability of data

All manuscripts must include a data availability statement. This statement should provide the following information, where applicable:

- Accession codes, unique identifiers, or web links for publicly available datasets
- A list of figures that have associated raw data
- A description of any restrictions on data availability

Accession codes, unique identifiers, or web links for publicly available datasets are listed in the manuscript.

Field-specific reporting

Please select the one below that is the best fit for your research. If you are not sure, read the appropriate sections before making your selection.

☒ Life sciences ☐ Behavioural & social sciences ☐ Ecological, evolutionary & environmental sciences

For a reference copy of the document with all sections, see nature.com/documents/nr-reporting-summary-flat.pdf

Life sciences study design

All studies must disclose on these points even when the disclosure is negative.

Sample size	N/A
Data exclusions	No data were excluded
Replication	Most experiments were repeated three times unless as indicated where a few repeated twice.
Randomization	We randomly took fluorescence confocal microscopic images.
Blinding	N/A

Reporting for specific materials, systems and methods

We require information from authors about some types of materials, experimental systems and methods used in many studies. Here, indicate whether each material, system or method listed is relevant to your study. If you are not sure if a list item applies to your research, read the appropriate section before selecting a response.

Materials & experimental systems

n/a	Involved in the study	
☐	☒	Antibodies
☐	☒	Eukaryotic cell lines
☒	☐	Palaeontology and archaeology
☒	☐	Animals and other organisms
☒	☐	Human research participants
☒	☐	Clinical data
☒	☐	Dual use research of concern

Methods

n/a	Involved in the study	
☒	☐	ChIP-seq
☐	☒	Flow cytometry
☒	☐	MRI-based neuroimaging

Antibodies

Antibodies used	ALDH1A3 antibody (cat# ab129815) from Abcam was used throughout. Beta-ACTIN (cat#A5441,clone AC-15) from Sigma-Aldrich was used as the loading controls. CPOX antibody (cat# NBP2-59438) from Novus Biologicals was used for confirming the knockout of CPOX and α-Actinin antibody (cat# 12413S) from Cell Signaling was used as the loading controls for the CPOX analysis.
Validation	All the antibodies are are commercially available and have been validated by the manufacturers. Detailed information can be found on the website from the manufacturers.<br>ALDH1A3 antibody (cat# ab129815): https://www.abcam.com/aldh1a3-antibody-ab129815.html<br>Beta-ACTIN (cat#A5441,clone AC-15): https://www.sigmaaldrich.com/US/en/product/sigma/a5441?context=product<br>CPOX antibody (cat# NBP2-59438): https://www.novusbio.com/products/cpox-antibody-36b10_nbp2-59438<br>α-Actinin antibody: https://www.cellsignal.com/products/antibody-conjugates/a-actinin-d6f6-xp-rabbit-mab-hrp-conjugate/12413

Eukaryotic cell lines

Policy information about cell lines

Cell line source(s)	Glioma stem cells (GSCs) were derived from high-grade glioma (HGG) samples, the MES subtype (GSC-83, GSC-326) and the PN subtype (GSC-19, GSC-84), were described by us previously (Mao et al. 2013). U87MG cells are from ATCC (ATCC® HTB-14™). 293FT cells (Cat# R70007) are from Thermo Fisher Scientific.
Authentication	GSCs are patient derived cells with no authentication. U87MG were authenticated. 293FT was used only for lentivirus production and did not undergo authentication.
Mycoplasma contamination	GSC-83, GSC-326 tested mycoplasma negative. U87MG tested by ATCC as mycoplasma negative. 293FT was tested mycoplasma negative. All cells in the Sobol lab are routinely tested for mycoplasma contamination (every three months).
Commonly misidentified lines (See ICLAC register)	*Name any commonly misidentified cell lines used in the study and provide a rationale for their use.*

Flow Cytometry

Plots

Confirm that:

☒ The axis labels state the marker and fluorochrome used (e.g. CD4-FITC).

☒ The axis scales are clearly visible. Include numbers along axes only for bottom left plot of group (a 'group' is an analysis of identical markers).

☒ All plots are contour plots with outliers or pseudocolor plots.

☒ A numerical value for number of cells or percentage (with statistics) is provided.

Methodology

Sample preparation	GSC-83, GSC-326 tested mycoplasma negative. U87MG tested by ATCC as mycoplasma negative. 293FT was tested mycoplasma negative.
Instrument	ALDH activity was analyzed by flow cytometry using a FACSCanto II (BD Biosciences, San Jose, CA). The cells were sorted by flow cytometry using a FACSAria III (BD Biosciences, San Jose, CA).
Software	Data acquisition and analysis was performed using Diva software version 8.3 (BD Biosciences, San Jose, CA)
Cell population abundance	Cell sorting experiments were performed to isolate the lowest and highest 10% of cells stained for ALDH activity. Post sort analysis was done to confirm purity of the selected sort gates.
Gating strategy	For ALDH analysis all intact cells were included in the base gate to exclude clumps and debris on FSC/SCC. The analysis gate was set on the negative DEAB control. Similarly cell sorting was performed in the same manner with the addition of two doublet gating histograms (FSC-W/FSC-A and SSC-W.SSC-A) to ensure single cell section for high purity sorting.

☒ Tick this box to confirm that a figure exemplifying the gating strategy is provided in the Supplementary Information.
